# BACE1 partial deletion induces synaptic plasticity deficit in adult mice

**DOI:** 10.1038/s41598-019-56329-7

**Published:** 2019-12-27

**Authors:** Sylvia Lombardo, Martina Chiacchiaretta, Andrew Tarr, WonHee Kim, Tingyi Cao, Griffin Sigal, Thomas W. Rosahl, Weiming Xia, Philip G. Haydon, Matthew E. Kennedy, Giuseppina Tesco

**Affiliations:** 1 Alzheimer’s Disease Research Laboratory, Department of Neuroscience, Tufts University School of Medicine, Boston, Massachusetts 02111 USA; 2 Department of Neuroscience, Tufts University School of Medicine, Boston, Massachusetts 02111 USA; 3 Circuits and Behaviour Core, Center for Neuroscience Research, Tufts University School of Medicine, Boston, Massachusetts 02111 USA; 4 External In Vivo Pharmacology, Merck & Co. Inc., Kenilworth, NJ 07033 USA; 5 Geriatric Research, Education and Clinic Center, Bedford Veterans Affairs Medical Center, Bedford, MA 01730 USA; 6 Department of Pharmacology and Experimental Therapeutics, Boston University School of Medicine, Boston, MA 02118 USA; 7 Department of Neuroscience, Merck & Co. Inc, Boston, MA 02115 USA

**Keywords:** Alzheimer's disease, Dementia

## Abstract

BACE1 is the first enzyme involved in APP processing, thus it is a strong therapeutic target candidate for Alzheimer’s disease. The observation of deleterious phenotypes in BACE1 Knock-out (KO) mouse models (germline and conditional) raised some concerns on the safety and tolerability of BACE1 inhibition. Here, we have employed a tamoxifen inducible BACE1 conditional Knock-out (cKO) mouse model to achieve a controlled partial depletion of BACE1 in adult mice. Biochemical and behavioural characterization was performed at two time points: 4–5 months (young mice) and 12–13 months (aged mice). A ~50% to ~70% BACE1 protein reduction in hippocampus and cortex, respectively, induced a significant reduction of BACE1 substrates processing and decrease of Aβx-40 levels at both ages. Hippocampal axonal guidance and peripheral nerve myelination were not affected. Aged mice displayed a CA1 long-term potentiation (LTP) deficit that was not associated with memory impairment. Our findings indicate that numerous phenotypes observed in germline BACE1 KO reflect a fundamental role of BACE1 during development while other phenotypes, observed in adult cKO, may be absent when partially rather than completely deleting BACE1. However, we demonstrated that partial depletion of BACE1 still induces CA1 LTP impairment, supporting a role of BACE1 in synaptic plasticity in adulthood.

## Introduction

Alzheimer’s disease (AD) is the most common type of dementia characterized by loss of memory and degradation of cognitive function. A key neuropathological event in AD aetiology is the accumulation of the amyloid-β (Aβ) peptide that originates from serial proteolysis of the amyloid precursor protein (APP). β-secretase, known as β-site APP cleaving enzyme 1 (BACE1), is the first enzyme involved in APP processing^1–6^. Thus, inhibiting BACE1 pharmacologically is a rational strategy for AD treatment^7–10^. Although BACE1 inhibition seems promising, many studies identified multiple phenotypes in germline BACE1 knock-out (BACE1^−/−^) mice such as axon guidance defects^11,12^, hypomyelination^13,14^, increased astrogenesis^15^, sensorimotor gating impairment^16^ and memory impairment^17^. While these findings shed some light on BACE1 function, they also raise concerns about the possible side effects of therapeutic BACE1 inhibition in AD patients. The observation that BACE1 expression level is highest during postnatal development in mice and decreases with age^13^ lead to the hypothesis that the phenotypes observed in the BACE1^−/−^ mice were caused by BACE1 function during development. Thus, BACE1 inhibition could be tolerated in adulthood. In addition, BACE1 KO in a mouse model of AD led to suppression of AD pathology and prevented cognitive impairment^17,18^.

To investigate the consequences of BACE1 inhibition in adulthood, conditional models (cKO) of BACE1 were recently developed^19,20^. These studies showed that BACE1 depletion in adult mice reverses amyloid deposition by reducing the amount of plaques in 5xFAD mice. However, an almost total BACE1 depletion (mimicking pharmacological inhibition at high doses) was shown to induce LTP and axonal guidance defects^19,20^. It is worth mentioning that both cited studies achieved an almost total depletion of BACE1 protein in the adult mice. We hypothesized that a lower level of BACE1 depletion may not result in negative phenotypes while still eliciting inhibition of CNS Aβ. To test this hypothesis, we achieved a partial deletion of BACE1 in a cKO mouse model and we characterized mice following both short-term acute depletion and long-term, prolonged depletion of BACE1. To do so, we treated mice BACE1^flox/flox^;RosaCreERT2^+/WT^ with tamoxifen (TAM). Mice were characterized at two time points: 4–5 months (young) and 12–13 months (aged) corresponding to 1 and 10 months after cessation of TAM treatment respectively. In both investigated cohorts we detected reduced processing of multiple BACE1 substrates and a 50% decrease in Aβx-40 levels. Hippocampal axonal guidance and peripheral nerve myelination were not affected, however in aged mice we observed an LTP deficit in the CA1 region of the hippocampus that was not associated with a memory impairment in the behavioural tests investigated (Y maze and contextual fear conditioning).

Our findings indicate that many of the phenotypes observed in BACE1^−/−^ mice reflect a fundamental role of BACE1 during development while other phenotypes, observed in adult cKO, may be absent when partially rather than completely inhibiting BACE1. However, we demonstrated that a partial depletion of BACE1 still induces CA1 LTP impairment, supporting a role of BACE1 in synaptic plasticity in adulthood.

## Results

### BACE1 is depleted in adult BACE1 cKO mice following tamoxifen treatment

**Figure 1:**
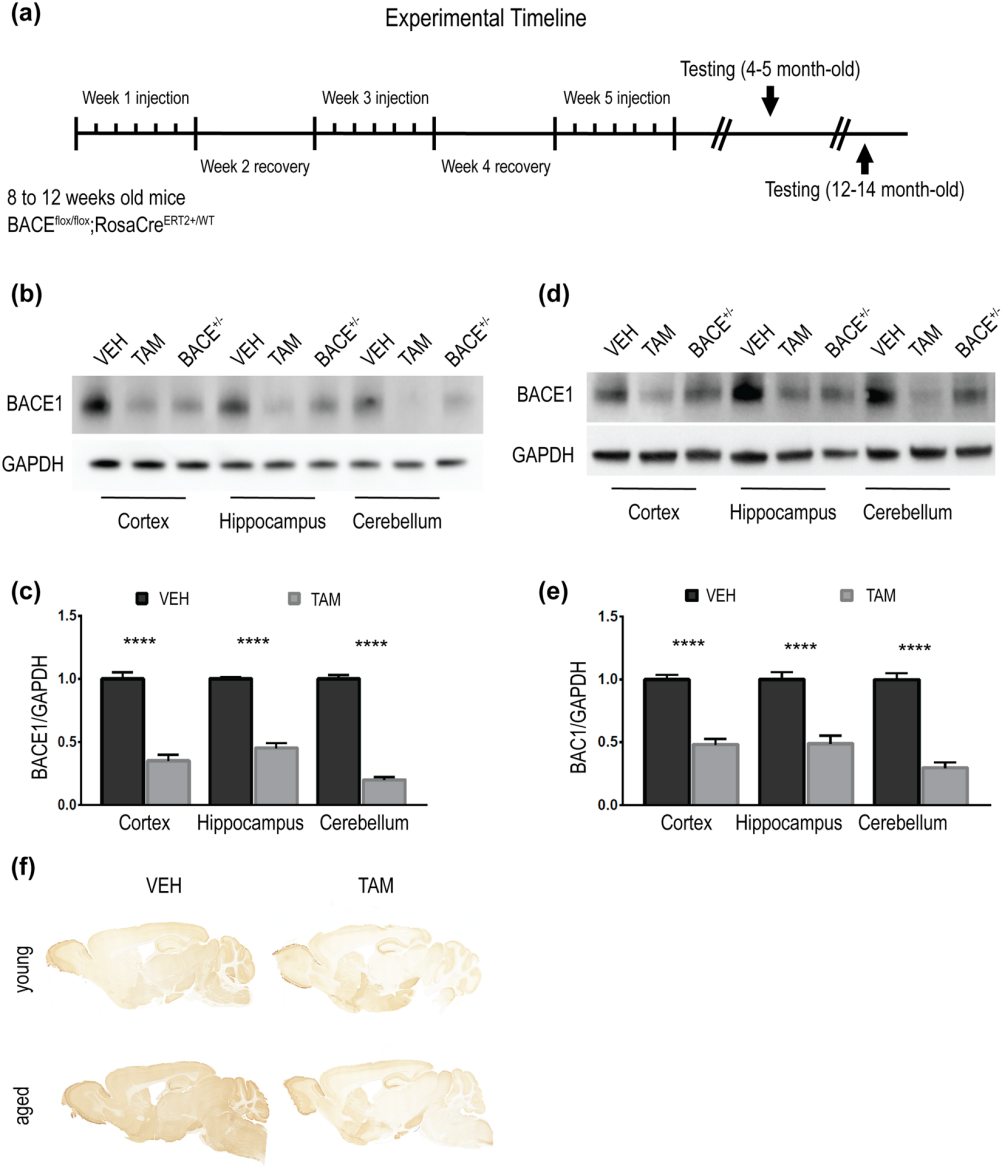
BACE1 is depleted in adult BACE1 cKO mice following tamoxifen treatment. (**a**) Experimental timeline showing treatment strategy used for tamoxifen or vehicle administration and time of experiments. Mice BACE1^flox/flox^;RosaCreERT2^+/WT^ between 8 and 12 weeks of age were treated with a daily dose of 200 mg/kg of body weight of tamoxifen (TAM-treated) or vehicle (VEH-treated) for five consecutive days across five weeks of treatment, every week of injection being followed by a week of recovery. Mice were tested at two different time points: 4–5 (young) and 12–14 (aged) months of age. Brain homogenates from cortex, hippocampus or cerebellum of TAM- or VEH-treated mice were resolved by SDS-PAGE for Western blot analysis of BACE1 expression level (D10E5). Homogenates from aged-matched BACE1^+/−^ were also loaded as control samples (not included in densitometry analysis). GAPDH (MAB374) was used as loading control. Panels (b,d) are representative blots of TAM- or VEH-treated mice collected at 4–5 and 12–13 months of age respectively. (**c**) Densitometry analysis of protein expression. BACE1 levels were normalized to GAPDH and expressed as arbitrary unit (A.U.). Protein amount in TAM-treated mice was normalized to protein levels in control mice (set at 1). The percentage of BACE1 decrease in TAM-treated mice was: ~70% in cortex, ~60% in hippocampus and ~80% in cerebellum in young mice (VEH n = 8; TAM n = 8). (**e**) Comparable results were observed in old mice (BACE1 decrease of ~50% in cortex, ~50% in hippocampus and ~70% in cerebellum) (VEH n = 7; TAM n = 7). (**f**) Representative images of immunohistochemistry of sagittal sections of TAM- or VEH-traded mice collected at 4–5 (young) and 12–13 (aged) months of age showing decrease expression of BACE1 (EPR3956) in TAM-treated mice. Results were plotted as Mean ± SEM, ****p < 0.0001, Student’s t test.

To investigate the effects of BACE1 depletion in adult mice we generated a BACE1 cKO model. The Exon2 of BACE1 gene was flanked by loxP sites by homologous recombination (BACE1^flox/flox^) (Supplementary Fig. 1a). Whole body inducible recombination was achieved by crossing the BACE1^flox/flox^ line with the Rosa26CreERT2 line^21^ obtaining mice homozygous for BACE1 floxed allele and hemizygous for the Rosa26CreERT2 allele (BACE1^flox/flox^;RosaCreERT2^+/WT^) (Supplementary Fig. 1b). To induce recombination mice (between 8 and 12 weeks of age) were treated with 200 mg/kg per body weight of TAM or vehicle (VEH) over a 5-week period consisting of 5 consecutive days of dosing in weeks 1, 3 and 5. Each treatment week was followed by a week of recovery (i.e. weeks 2 and 4; Fig. 1a). Mice were analysed at two different intervals (Fig. 1a), 4–5 month-old (young), corresponding to 1 month after treatment cessation and 12–13 month-old (aged), corresponding to 10 months after treatment. BACE1 mRNA levels were decreased in cortex, hippocampus and cerebellum of young TAM-treated mice compared to controls (Supplementary Fig. 2) (VEH-cortex:1; TAM-cortex:0.31; VEH-hippocampus:1, TAM-hippocampus:0.50; VEH-cerebellum:1; TAM-cerebellum:0.32; expressed as fold-change; VEH n = 9; TAM n = 9; p < 0.0001). BACE1 protein expression was decreased in young TAM-treated mice and differed in the areas investigated, ~70% in cortex, ~60% in hippocampus and ~80% in cerebellum (Fig. 1b,c) (VEH-cortex:1 ± 0.05; TAM-cortex:0.35 ± 0.04; VEH-hippocampus:1 ± 0.01; TAM-hippocampus:0.45 ± 0.03; VEH-cerebellum:1 ± 0.03; TAM-cerebellum:0.19 ± 0.02; VEH n = 8, TAM n = 8; p < 0.0001). Samples from BACE1^−/+^ mice were also run for comparison with samples from TAM-treated mice. To establish whether TAM itself could affect BACE1 protein levels, we treated BACE1^flox/flox^ mice (not carrying RosaCreERT2 allele) with the same protocol of drug administration (Fig. 1a) and euthanized them at 4–5 months of age (one month after the TAM treatment). Protein level of BACE1 were not altered in cortex of BACE1^flox/flox^ TAM-treated compared to untreated WT mice, indicating that the decrease in BACE1^flox/flox^;RosaCreERT2^+/WT^ was due to the recombination (Supplementary Fig. 3) (BACE1^flox/flox^:0.91 ± 0.03, n = 6; WT:1 ± 0.08, n = 5; ns). Next, we determined BACE1 expression levels in aged mice. A significant reduction of BACE1 expression was observed: ~50% in cortex, ~50% in hippocampus and ~70% in cerebellum (Fig. 1d,e), (VEH-cortex:1 ± 0.03; TAM-cortex:0.48 ± 0.04; VEH-hippocampus:1 ± 0.05; TAM-hippocampus:0.48 ± 0.06; VEH-cerebellum:1 ± 0.04; TAM-cerebellum:0.29 ± 0.04; VEH n = 7; TAM n = 7; p < 0.0001). Immunohistochemistry confirmed decreased levels of BACE1 in brain sections of TAM-treated mice compared to controls at both time points (Fig. 1f). Depletion of BACE1 did not affect the protein expression level of APP-metabolism related genes, ADAM Metallopeptidase Domain 10 (ADAM10) and Presenilin 1 (PS1), α- and γ-secretase respectively (ADAM10: VEH:1 ± 0.01, TAM:1.08 ± 0.06; PS1: VEH:1 ± 0.03, TAM:1.01 ± 0.03, n = 8; ns) (Supplementary Fig. 4).

### BACE1-mediated processing of APP and CHL1 is reduced in both young and aged BACE1 cKO mice following tamoxifen treatment

**Figure 2:**
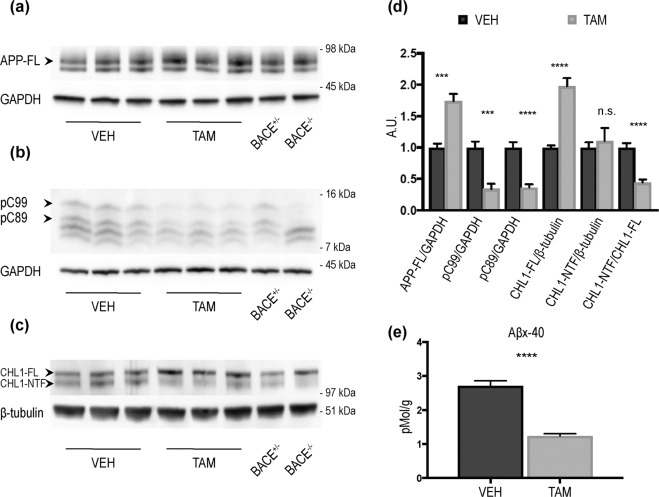
BACE1-mediated processing of APP and CHL1 is reduced in cortex of young BACE1 cKO mice following tamoxifen treatment. Cortex homogenates from TAM- or VEH-treated mice were resolved by SDS-PAGE for Western blot analysis of APP and CHL1 processing. Homogenates from aged-matched BACE^+/−^ and BACE1^−/−^ were also loaded as control samples. Representative blots of (**a**) APP-full length (APP-FL) (C1/6.1), (**b**) APP-Carboxy Terminal Fragments (CTFs) (C1/6.1) and (**c**) CHL1. (**d**) Densitometry analysis of protein expression. Protein amount was normalized to protein levels in control mice (set at 1). APP-FL, pC99 and pC89 were normalized to GAPDH (MAB374) while CHL1-FL and CHL1-NTF were normalized to β-tubulin (JDR.3B8). APP processing was reduced in TAM-treated mice as demonstrated by the accumulation of APP-FL (C1/6.1), and reduced levels of the βCTFs pC99 and pC89. βCTFs were clearly identified because missing in the BACE1^−/−^ sample. CHL1-FL (AF2147) levels were increased while CHL1-N Terminal Fragment (CHL1-NTF) levels were not affected in cortex of TAM-treated mice. However, the CHL1-NTF/CHL1-FL ratio was significantly decreased in TAM-treated mice demonstrating reduced BACE1 processing (VEH n = 8; TAM n = 8). (**e**) Aβx-40 was quantified from brain homogenates by ELISA (VEH n = 8; TAM n = 8). Levels of Aβx-40 expressed as pMol/g of cortex were significantly reduced in TAM-treated mice (~50% decrease). Results were plotted as Mean ± SEM, ***p < 0.001; ****p < 0.0001; n.s. = not significant, Student’s t test.

**Figure 3:**
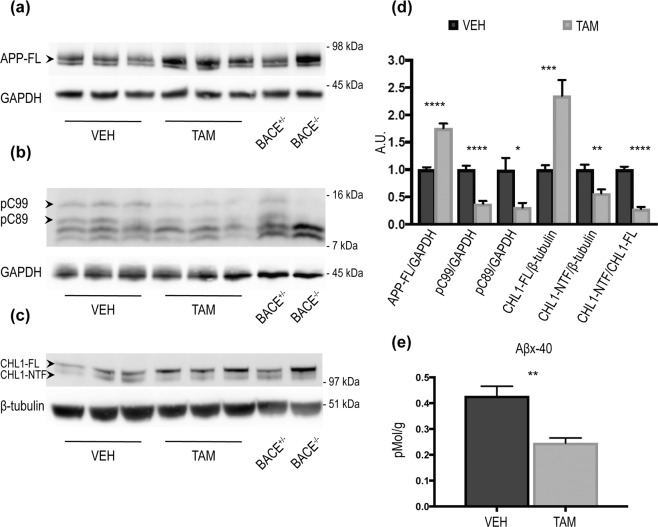
BACE1-mediated processing of APP and CHL1 is reduced in cortex of aged BACE1 cKO mice following tamoxifen treatment. Cortex homogenates from TAM- or VEH-treated mice were resolved by SDS-PAGE for Western blot analysis of APP and CHL1 processing. Homogenates from aged-matched BACE^+/−^ and BACE1^−/−^ were also loaded as control samples. APP-FL, pC99 and pC89 were normalized to GAPDH (MAB374) while CHL1-FL and CHL1-NTF were normalized to β-tubulin (JDR.3B8). Protein amount was normalized to protein levels in control mice injected with vehicle (set at 1). Representative blots of (**a**) APP-FL (C1/6.1), (**b**) APP-CTFs (C1/6.1) and **(c**) CHL1. (**d**) Densitometry analysis of protein expression. APP processing was reduced in TAM-treated mice as demonstrated by the accumulation of APP-FL (C1/6.1), and reduced levels of the βCTFs pC99 and pC89. βCTFs were clearly identified because missing in the BACE1^−/−^ sample. CHL1-FL (AF2147) levels were increased and CHL1-NTF levels were significantly reduced. Furthermore, the CHL1-NTF/CHL1-FL ratio was significantly decreased in TAM-treated mice demonstrating reduced BACE1 processing (VEH n = 7; TAM n = 7). (**e**) Quantification of Aβx-40 was performed by MSD immunoassay on cortex homogenates and expressed as pMol/g of cortex. The decrease of levels of Aβx-40 in TAM-treated mice was comparable to the one observed in samples collected from young TAM-treated mice (~50% decrease) (VEH n = 7; TAM n = 7). Results were plotted as Mean ± SEM, *p < 0.05; **p < 0.005; ***p < 0.001; ****p < 0.0001; n.s. = not significant, Student’s t test.

Next, we investigated how reduced levels of BACE1 affected the processing of multiple substrates. Cortex homogenates of TAM- and VEH-treated mice were analysed. Western blot analysis showed an accumulation of APP-full length in young mice (APP-FL) (VEH:1 ± 0.06, n = 7; TAM:1.73 ± 0.11, n = 8; p < 0.001) (Fig. 2a,d) corresponding to decreased levels of phosphor-C89 (pC89) (VEH:1 ± 0.08, n = 8; TAM:0.36 ± 0.05 n = 8; p < 0.0001) and phosphor-C99 (pC99) (VEH:1 ± 0.09, n = 8; TAM:0.34 ± 0.07, n = 8; p < 0.001) (Fig. 2b,d). We then quantified the processing of CHL1, because of its high affinity for BACE1^22,23^. CHL1-full length was accumulated in cortex homogenates (CHL1-FL) (Fig. 2c,d) (VEH:1 ± 0.03, n = 8; TAM:1.97 ± 0.13, n = 8; p < 0.0001), while levels of CHL1-N Terminal Fragment (CHL1-NTF) (Fig. 2c,d) (VEH:1 ± 0.08, n = 8; TAM:1.10 ± 0.20, n = 8; ns) were not affected consistent with CH-L1 proteolysis by multiple sheddases. However, the overall processing of CHL1 was impaired as indicated by a decrease in the CHL1-NTF/CHL1-FL ratio (Fig. 2d) (VEH:1 ± 0.06, n = 8; TAM:0.44 ± 0.04, n = 8; p < 0.0001). To further characterize BACE1 CNS activity following recombination we quantified the level of Aβx-40 in cortical samples from VEH- or TAM-treated mice by ELISA. We observed ~50% decrease of Aβx-40 levels in TAM-treated mice (Fig. 2e) (VEH:2.712 ± 0.1506, n = 8; TAM:1.237 ± 0.06744, pMol/g, n = 8; p < 0.0001). The analysis of cortical samples collected from aged TAM-treated mice revealed comparable results. Briefly, APP-FL was increased (Fig. 3a,d) (VEH:1 ± 0.04, n = 7; TAM:1.75 ± 0.08, n = 7; p < 0.0001), corresponding to decreased levels of pC89 (VEH:1 ± 0.22, n = 7; TAM:0.31 ± 0.08, n = 7; p < 0.05) and pC99 (VEH:1 ± 0.07, n = 7; TAM:0.37 ± 0.04, n = 7; p < 0.0001) (Fig. 3b,d). CHL1 processing was also impaired as demonstrated by the accumulation of the CHL1-FL (Fig. 3c,d) (VEH:1 ± 0.07, n = 7; TAM:2.35 ± 0.28, n = 7; p < 0.001). Lower levels of CHL1-NTF were also observed in this cohort (Fig. 3a,d) (VEH:1 ± 0.09, n = 7; TAM:0.56 ± 0.07, n = 7; p < 0.01). Aβx-40 quantified by MSD immunoassay was decreased in TAM-treated vs. VEH-treated mice (~50%) (Fig. 3e) (VEH:0.42 ± 0.03, n = 7; TAM:0.24 ± 0.01, pMol/g, n = 7; p < 0.01).

### Sciatic nerve myelination was not altered in young and aged BACE1 cKO mice following tamoxifen treatment

**Figure 4:**
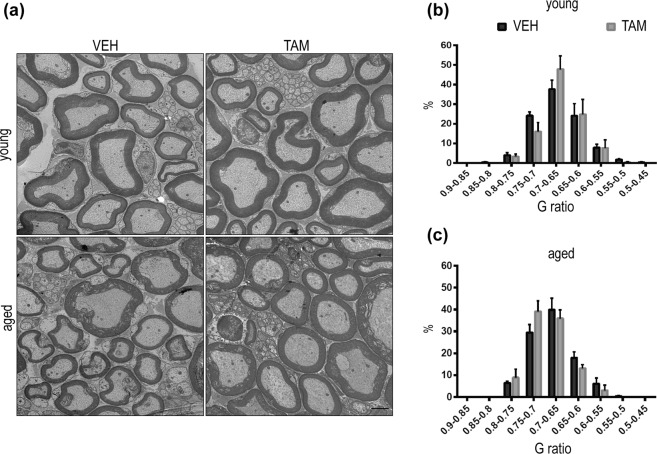
Sciatic nerve myelination was normal in young and aged BACE1 cKO mice following tamoxifen treatment. (**a**) Electron microscopy representative images of sciatic nerves from TAM- or VEH-treated mice, collected from 4–5 (upper panel) and 12–13 month (lower panel) old mice. Scale bar 2 μm. G-ratio analysis showed no myelination alteration in TAM-treated group compared to controls in young (**b**) or aged (**c**) mice (VEH n = 3; TAM n = 3, from 100 to 150 fibers were analyse from each mouse). Data were plotted as g ratio in function of fibers’ frequency ± SEM, Mann-Whitney test and Kolmogorov-Smirnov test.

In light of the hypomyelination phenotype observed in BACE1^−/−^mice^13,14^ we quantified peripheral nerve myelination in our cohort of mice in which we achieved partial BACE1 depletion in adulthood. Sciatic nerves from young and aged mice TAM- or VEH-treated were collected. G-ratio quantification of electron microscopy images (Fig. 4a) was performed and no myelination deficit was observed in young (Fig. 4b) (Mann-Whitney p = 0.61, ns; Kolmogorov-Smirnov test p = 0.7, ns; VEH n = 3; TAM n = 3) and aged mice treated with TAM (Fig. 4c) (Mann-Whitney p = 0.85, ns; Kolmogorov-Smirnov test p > 0.99, ns; VEH n = 3; TAM n = 3) indicating that the hypomyelination phenotype is most likely due to BACE1 activity during the development.

### Normal axon guidance in hippocampus mossy fibers of aged BACE1 cKO mice following partial BACE1 deletion

**Figure 5:**
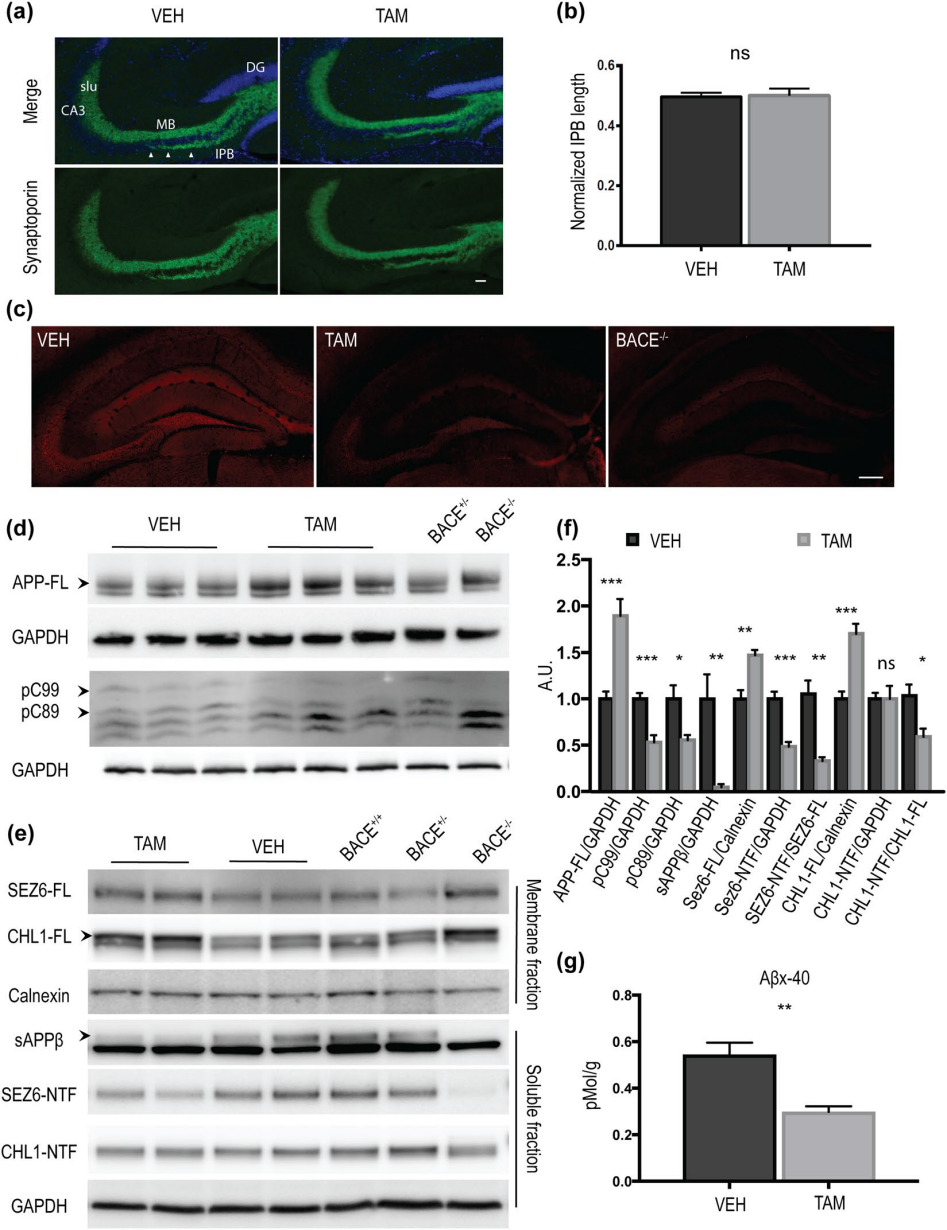
Axon guidance defects were absent in hippocampus mossy fibers of aged BACE1 cKO mice following partial BACE1 deletion. (**a**) Coronal sections collected from aged mice were stained with anti-synaptoporin (SPO) antibody (green) and DAPI (blue). Scale bar 50 μm. (**b)** Quantification of IPB length showed no alteration in TAM-treated mice compared to controls. IPB length was normalized on the length of the CA3 stratum lucidum (VEH n = 8; TAM n = 7, 3 to 4 sections per mouse). (**c**) Representative microscopy images showing reduced BACE1 (D10E5) expression in the hippocampus of TAM-treated mice. BACE1 signal was totally absent in BACE^−/−^ mice, used as control to evaluate the amount of background in the staining. Scale bar 200 μm. Hippocampus full homogenates from TAM- or VEH-treated mice were resolved by SDS-PAGE for analysis of APP processing and fractionated (soluble and membrane fractions) for the analysis of SEZ6 and CHL1 processing. Homogenates from aged-matched BACE^+/−^ and BACE1^−/−^ were loaded as control samples. Representative blots of (**d**) APP-FL (C1/6.1) and APP- CTFs (C1/6.1), (**e**) fractionation blots of sAPPβ (BAWT), SEZ6 (14E5) and CHL1 (AF2147). (**f**) Densitometry analysis of protein expression. APP processing was reduced in TAM-treated mice as demonstrated by the accumulation of APP-FL (C1/6.1), and reduced levels of the βCTFs pC99 and pC89, and sAPPβ. βCTFs and sAPPβ were identified because missing in the BACE1^−/−^ sample. SEZ6 processing was decreased in TAM-treated mice with accumulation of the full length and decreased levels of the ectodomain (SEZ6-NTF) as well as decreased SEZ6-NTF/SEZ6FL ratio. Processing of CHL1 was also impaired as showed by increased of CHL1-FL levels, while CHL1-NTF was not altered. CHL1-NTF/CHL1-FL ratio was significantly decreased. APP-FL, CTFs, SEZ6-NTF and CHL1-NTF were normalized to GAPDH (MAB374), SEZ6-FL and CHL1-FL were normalized to Calnexin (610523) (VEH n = 5; TAM n = 5). (**g**) Aβx-40 was quantified from hippocampus homogenates by MSD immunoassay. TAM-treated group displayed a significant reduction of Aβx-40 levels (~50% decrease) compared to control (VEH n = 7; TAM n = 7). Results were plotted as Mean ± SEM, **p < 0.005; ***p < 0.001; n.s. = not significant, Student’s t test. DG: dentate gyrus, IPB: infrapyramidal bundle, slu: stratum lucidum, MB: main bundle.

Previous studies have shown that germline deletion of BACE1 results in axon guidance defects in the hippocampus and olfactory bulb^12^. Recently, Ou-Yang *et al*.^19^ reported the presence of the same phenotype in a BACE1 cKO model in which a ~90–95% depletion of BACE1 was achieved in cortex and hippocampus of 12 month-old mice. Thus, we performed a morphological study of the infrapyramidal bundle (IPB) in the hippocampus of aged TAM-treated mice in which BACE1 levels are ~50% less than VEH-treated mice (Fig. 1). Brain sections from mice treated with VEH or TAM were immunostained with anti-synaptoporin (SPO) antibody (Fig. 5a) and the length of the IPB was measured (Fig. 5b). The length of the IPB was not altered in TAM-treated compared to VEH-treated mice (VEH:0.49 ± 0.01, n = 8; TAM:0.50 ± 0.02, n = 7, µm: IPB/MB + slu; ns). Reduced BACE1 expression level in the hippocampus of TAM-treated mice was confirmed by immunostaining (Fig. 5c). As a control, brain sections of BACE1^−/−^ mice were also stained. We confirmed the shortening of IPB in BACE1^−/−^ mice (Supplementary Fig. 5). Next, we investigated the processing of BACE1 substrates in hippocampus homogenates from aged TAM- or VEH-treated mice. We observed an accumulation of APP-FL (VEH:1 ± 0.08, n = 7; TAM:1.89 ± 0.18, n = 7; p < 0.001) and a decrease of the βCTFs pC89 (VEH:1 ± 0.14, n = 7; TAM:0.55 ± 0.05, n = 7; p < 0.05) and pC99 (VEH:1 ± 0.06, n = 7; TAM:0.53 ± 0.07, n = 7; p < 0.001) (Fig. 5d). Since CHL1 processing impairment was previously associated to axon guidance defects^12,24^ we performed fractionation of hippocampus samples to achieve a better separation of CHL1-FL and CHL1-NTF. Membrane and soluble fractions (Fig. 5e) analysis revealed CHL1-FL accumulation (VEH:1 ± 0.08, n = 5; TAM:1.70 ± 0.10, n = 5, p < 0.001), while CHL1-NTF level was not affected in TAM-treated mice (VEH:1 ± 0.06, n = 5; TAM:1.00 ± 0.13, n = 5; ns) (Fig. 5f). We then investigated levels of SEZ6, which is exclusively processed by BACE1^23,25^. Membrane fraction analysis revealed SEZ6-FL accumulation (VEH:1 ± 0.09, n = 5; TAM:1.47 ± 0.05, n = 5; p < 0.01), decreased SEZ6-NTF levels in the soluble fraction (VEH:1 ± 0.07, n = 5; TAM:0.48 ± 0.05, p < 0.05, n = 5) and decreased SEZ6-NTF/SEZ6-FL ratio (Fig. 5e,f). Moreover, TAM-treated mice displayed very low levels of APP soluble β fragment (sAPPβ) (VEH:1 ± 0.26, n = 5; TAM:0.04 ± 0.03, n = 5). Aβx-40 levels, quantified by MSD immunoassay, were found to be decreased in hippocampus of aged TAM-treated mice (~50%decrease) (Fig. 5g) (VEH:0.54 ± 0.05, n = 7 TAM:0.29 ± 0.02, pMol/g, n = 7; p < 0.01).

### LTP deficit is induced in aged BACE1 cKO mice following partial BACE1 depletion

**Figure 6:**
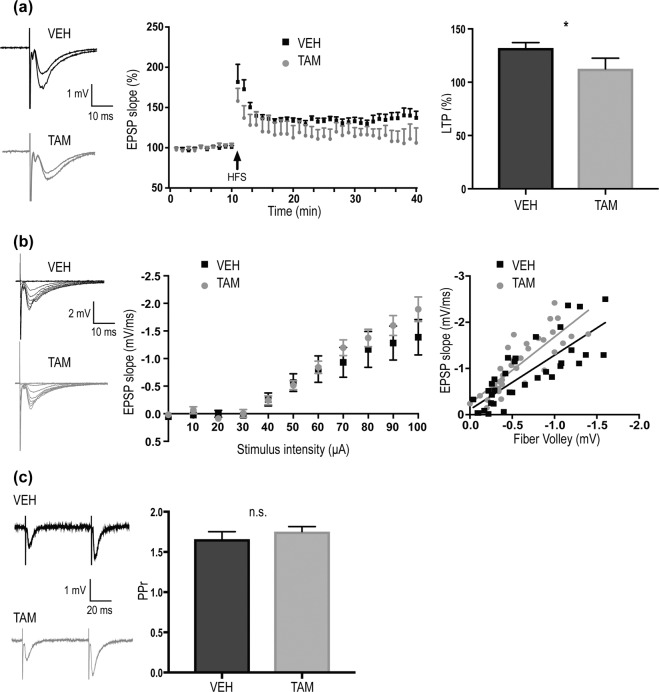
LTP deficit is induced in aged BACE1 cKO mice following partial BACE1 depletion. (**a**) Left: Representative traces for VEH and TAM-treated mice at baseline and after high frequency tetanic stimulation (HFS). Middle: Time course of normalized fEPSP slopes to the mean slope recorded during 10 min period before high-frequency tetanic stimulation (HFS), values were shown as average per minute for clarity. Right: The extent of LTP was calculated as a percentage of the baseline in the last 10 min of recording (VEH: 135.6 ± 6.928%, n = 10 from 4 mice; TAM: 112.5 ± 10.09%, n = 8 from 4 mice; p < 0.05). (**b**) Left: Representative traces of fEPSPs generated by gradually increasing stimulus intensity for VEH (black) and TAM (gray) treated mice. Middle: Input-output curve of fEPSP slope versus stimulus intensity. No differences were observed between the two groups (VEH and TAM: n = 5 slices from 2 mice). Right: Input–output curve relating the slope of the fEPSP to the presynaptic fiber volley amplitude, each point represents data from individual slices. No significative statistically differences were found, (VEH and TAM: n = 5 slices from 2 mice; p = 0.272). (**c**) Representative traces of fEPSPs recorded in CA1, in response to Schaffer collateral paired-pulse stimuli at 100 ms interstimulus interval (ISI) for VEH (black trace) and TAM (gray trace) treated mice. Bar graph showed facilitation ratio for the two conditions. No significative statistically differences were found (VEH: 1.66 ± 0.092, n = 8 slices from 2 mice; TAM: 1.753 ± 0.063, n = 9 slices from 2 mice; p = 0.357). Data were plotted as mean ± SEM. *p < 0.05; ns = not significant. Mann–Whitney U-test.

Genetic deletion^17,26^ and pharmacological inhibition of BACE1 in adult mice^27^, was shown to induce LTP deficit. However, whether LTP is impaired in cKO models remains controversial^19,20^. To address this question, field excitatory postsynaptic potentials (fEPSPs) were recorded in stratum radiatum in response to electrical stimulation of Shaffer Collateral fibers, in aged TAM- and VEH-treated mice. TAM-treated mice exhibited attenuated LTP in comparison to VEH-treated mice (VEH:132.1 ± 5.05, n = 12 TAM:112.5 ± 0.1, % from baseline, n = 11; p < 0.05) (Fig. 6a). LTP impairment was attributable to BACE1 partial depletion, since no deficit was observed in TAM-treated BACE1^flox/flox^ (not expressing the RosaCre allele), compared to BACE1^flox/flox^ uninjected controls (data not shown). Paired-pulse ratio (PPr), or input output curves were not affected, ruling out an effect on glutamate release probability and axonal excitability (Fig. 6b,c). These findings suggest that ~50% decrease of BACE1 protein in hippocampus is sufficient to affect synaptic plasticity, as detected by LTP impairment in the CA1 Shaffer collateral pathway.

### No behavioural deficits were detected in young and aged cKO mice following partial BACE1 deletion

**Figure 7:**
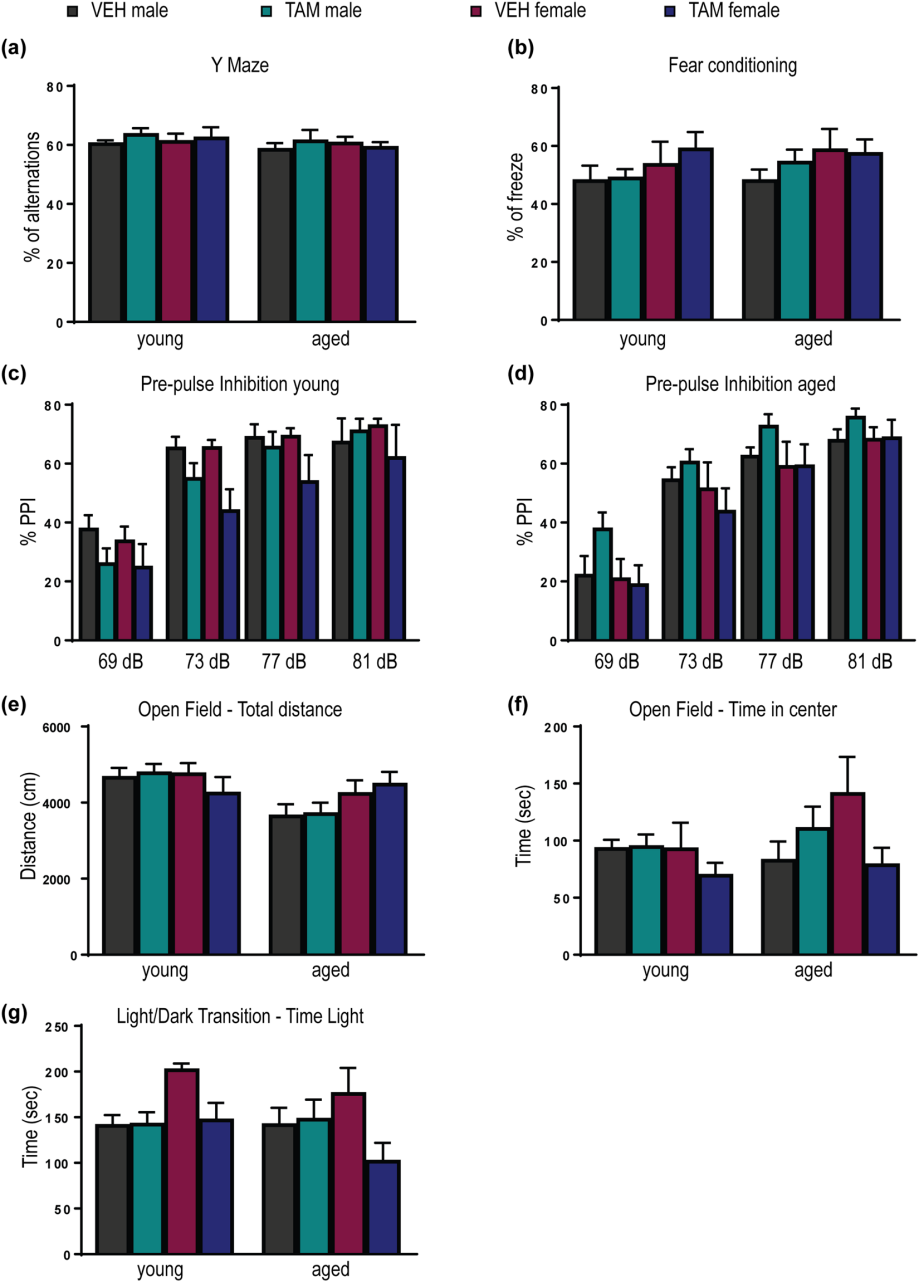
No behavioural deficits were detected in young and aged cKO mice following partial BACE1 deletion. TAM- or VEH-treated mice were subjected to a panel of behavioural tasks at 4–5 month-old (young). After aging, the same cohorts of mice were tested again at 12–13 months of age (aged) in the same set of behavioural tasks. Spontaneous alternation memory was investigated by Y maze task (**a**), while hippocampal dependent contextual memory was investigated by the contextual fear-conditioning paradigm (**b**). No memory deficit was detected in the investigated tasks. Pre-pulse Inhibition was performed to investigate the suppression of the acoustic startle response, both young (**c**) and aged mice (**d**) behaved normally. Open Field task showed no effect of the treatment in the total distance travelled (**e**), although the 3-way ANOVA analysis revealed a sex difference. Time spent in the center of the arena was also plotted to investigate anxiety phenotype, although a trend in TAM-treated females was observed at both ages, the data did not reach statistical significance (**f**). To further investigate the anxiety-like phenotype mice were subjected to the light/dark transition task, no difference was detected in the time spent exploring the lit compartment (**g**). Data were plotted as mean ± SEM. Analysis was performed by 3-way ANOVA repeated measure (time, treatment and sex used as independent variables), when no sex difference was detected male and female mice were pooled together and data were analysed by 2-way repeated measure ANOVA (time and treatment used as independent variables). See Supplementary Table 1 for p values (VEH male n = 13, TAM male n = 14, VEH female n = 6, TAM female n = 7).

Finally, we performed a panel of behavioural experiments on young and aged mice focusing on behavioural phenotypes that were shown to be altered in BACE^−/−^ mice such as memory, pre-pulse inhibition (PPI), locomotion and anxiety^16–18,28^. Y-maze and contextual fear conditioning were performed to investigate spontaneous alteration and hippocampal dependent memory respectively. No memory deficits were observed in young or aged TAM-treated mice compared to controls (Fig. 7a,b; Supplementary Table 1). Deficits in sensory motor gating have been previously reported in BACE1^−/−^ mice using the PPI task^16^. In this paradigm, a preceding low intensity tone (the pre-pulse) inhibits the reaction (startle response) to a higher intensity acoustic stimulus (the pulse). No deficit in TAM-treated mice was detected in both young and aged group when compared to VEH-treated mice (Fig. 7c,d; Supplementary Table 1). To assess locomotor activity and anxiety-like behaviour, mice were subjected to the open field test. The total distance travelled did not reveal any locomotor activity alteration in both young and aged groups; however, an effect of time was observed in male mice, aged male mice explored less (both TAM-treated and VEH-treated) compared to young male mice (p < 0.001) (Fig. 7e; Supplementary Table 1). Analysis for exploration time of the open field revealed no difference between TAM- and VEH-treated mice at both ages (Fig. 7f; Supplementary Table 1). To further evaluate the anxiety-like behavioural phenotype, mice were subjected to the light/dark transitions task. No anxiety-like behavioural phenotype was observed in both young and aged TAM-treated mice (Fig. 7g; Supplementary Table 1). Taken together these data support the hypothesis that a partial depletion of BACE1 does not induced behavioural deficits in the investigated tasks after acute short-term depletion (young animals) or long-term depletion (aged animals).

## Discussion

Emerging data from the literature indicate that a near total BACE1 deletion in adult mice induces alterations such as axonal guidance and LTP defects raising concerns for negative impacts on cognitive function under high levels of BACE inhibition achieved in clinical trials^29,30^. We hypothesized that a partial BACE1 deletion may be able to achieve meaningful reductions in CNS Aβ levels without eliciting deleterious phenotypes observed in previous models BACE1KO models.

To investigate the impact of BACE1 partial depletion on brain function we developed a unique cKO model that mimics partial BACE1 inhibition. TAM-treated BACE1^flox/flox^;RosaCreERT2^+/WT^ mice were analysed at young and old age, i.e. after short-term BACE1 depletion, or long-term BACE1 protein depletion. Though the degree of BACE1 decrease varied by brain region, the reduction was stable over time (Fig. 1c,e) mimicking a sustained level of Aβ lowering described for once daily dosing of verubecestat in AD patients^31^. The observed differences in recombination efficiency across different brain areas, are most likely due to variable tissue penetration of TAM^32^. At both young and old ages, processing of multiple validated BACE1 substrates was reduced (CHL1-NTF/CHL1-FL: ~30–40% reduction in cortex of young and aged mice, ~40% reduction in hippocampus of aged mice, SEZ6-FL/sSEZ6: ~70% reduction in hippocampus of aged mice). Moreover a~50% reduction of Aβx-40 levels were detected in both cortex and hippocampus (Figs. 2, 3 and 5), while sAPPβ levels were almost undetectable in hippocampus of aged mice (~90% reduction) (Fig. 5e,f). A direct correlation between Aβ and sAPPβ levels is expected when Aβ1–40 is measured. However, there is no commercially Aβ ELISA Aβ 1–40 for rodent is available. Thus, the difference in levels of Aβ and sAPPβ inhibition that we observed is most likely due to both the detection of N-terminally truncated Aβ peptides generated in absence of BACE1^33,34^ and the reduced sensitivity of detecting sAPPβ via western blotting.

To better characterize the impact of partial BACE1 depletion, we investigated phenotypes shown to be altered in BACE^−/−^mice. We quantified sciatic nerve myelination, and we found no deficit (Fig. 4) consistent with this phenotype arising from a developmental role of BACE1^13^. It is controversial whether BACE1 activity facilitates or delays remyelination events after nerve crush in adult mice^35,36^ and we cannot exclude that BACE1 inhibition might have consequences on remyelination events following injury in humans.

We then evaluated hippocampal structure and function phenotypes such as axon guidance and LTP. Previous studies identified axon guidance defects in the hippocampus of BACE^−/−^ mice^12^ and in BACE1cKO mice following 90–95% depletion in adult mice^19^. In our experimental design we retained ~50% expression of BACE1 in hippocampus of aged TAM-treated mice, and no axon guidance deficit was observed (Fig. 5a,b). Although the mechanism leading to disorganization of IPB in BACE1^−/−^ mice has not been elucidated, BACE1-mediated processing of CHL1 was hypothesized to be required for proper axon guidance in the dentate gyrus. In our experimental model, CHL1-FL accumulated, however, CHL1-NTF levels were not affected in TAM-treated mice compared to controls (Fig. 5e,f). The change in CH^-^L1-NTF suggests that additional sheddases (e.g. ADAM family) may substitute for BACE1-mediated cleavage. Thus, our data demonstrates that ~50% depletion of BACE1 does not elicit axon guidance deficits in dentate gyrus. It will be important to determine if varying degrees of pharmacological inhibition of BACE1 from ~50% to >90% have similar profiles.

Measurements of the LTP response in Schaffer collateral-CA1 synapses revealed LTP impairment in aged TAM-treated mice (Fig. 6). However, PPr was not affected at 100 ms interstimulus interval (ISI) in TAM-treated mice, indicating that the synaptic vesicle release probability was not impaired at this ISI. These data support the hypothesis that BACE1 modulates synaptic plasticity in adulthood. Dose-dependent LTP deficits in CA1 were previously reported in mice treated with BACE inhibitors, these deficits were associated with a reduction in spine density in the layer V pyramidal neurons in the somatosensory cortex^27,37^. However it is controversial whether BACE1 cKO display LTP deficit, Hu *et al*. observed reduced LTP following almost total BACE1 depletion in adult mice^20^ while LTP was reported to be normal in a separate BACE1 cKO where TAM treatment achieved 90%-95% reduction in BACE1 over 9 months^19^. BACE1 is located at both pre- and post-synaptic terminals^38,39^ and its substrates support both dendritic and axonal function. For example, SEZ6 is exclusively processed by BACE1 in mice and is required for normal dendritic arborization. SEZ6 null mice display reduced spine density and increased dendritic branching in cortical neurons^40^. In addition, it has been shown that chronic pharmacological inhibition of BACE impairs LTP in CA1 neurons in wild type but not in SEZ6 null mice, suggesting that BACE1-mediated processing of SEZ6 is required for synaptic plasticity^8^. Moreover, BACE1 can modulate axonal guidance and growth cone collapse that may be related to the processing of CHL1^11,12,41^. Taken together, these studies demonstrate the role of BACE1 in maintaining both dendritic and axonal morphology and function. Further studies are required to determine whether the LTP deficit observed here in BACE1 cKO TAM-treated mice could be associated with an alteration of axons and/or dendritic spines in CA1 or whether mossy fiber LTP is also affected. Despite the presence of an LTP deficit, no cognitive or behavioural alterations were observed in BACE1 cKO TAM-treated mice in the test that we investigated (Fig. 7).

Our study has some limitations and some questions remain to be addressed. In our model ~50% to ~70% BACE1 protein reduction resulted in an ~50% decrease of Aβx-40 in cortex and hippocampus, it remains to be determined if this reduction in Aβ would be enough to reverse amyloid plaque deposition, this can be achieved by crossing BACE1 cKO mice with murine models of AD or chronically administering a BACE inhibitor at ≤ED_50_. Studies from human genetics identified a mutation in the coding region of APP (A673T) in an Icelandic family that is protective against AD and cognitive decline^42^. This genetic variant results in decreased BACE1 processing of APP and reduced aggregation of Aβ42^43^. Plasma concentration of Aβ40 and Aβ42 in carriers of the A673T variant, were shown to be ~28% lower compared to controls^44^. These data demonstrate that a small reduction of Aβ throughout a life span is associated with protection against AD, suggesting that low level BACE1 inhibition could be useful as a long-term therapeutic approach.

To summarize, we achieved a partial genetic depletion of BACE1 that mimics an intermediate pharmacological inhibition, we demonstrated that most of the phenotypes observed in BACE^−/−^ mice and in BACE1 cKO after complete depletion of BACE1 are absent, however, an LTP deficit is still present when decreasing BACE1 expression to 50%. Importantly the deficit in LTP did not elicit behaviour impairments.

Previous studies in cKO mice demonstrated that an almost total BACE1 deletion could elicit adverse phenotypes, notably in the hippocampus. Although it is difficult to predict how the results obtained in animal models (axon guidance deficit, LTP) will translate to humans, however findings in BACE1 cKO mice suggest that high levels of BACE inhibition might have adverse effect on hippocampal functions such that any benefits coming from the Aβ decrease might be masked by adverse effects due to BACE1 inhibition during clinical trials.

Clinical trials based on the BACE inhibitor verubecestat (EPOCH and APECS) were recently discontinued^29,30^. As a result of verubecestat treatment, 60% (12 mg) to 75% (40 mg) average reduction of Aβ in cerebrospinal fluid was observed in association with regression of amyloid PET SUVR indicating that verubecestat was effective in lowering Aβ levels. However, a modest worsening of cognitive function was observed in prodromal AD subjects after 13 weeks of treatment with the inhibitors compared to placebo group. The cognitive impairment did not progress across the remainder of the 2-year trial period suggesting it may reflect an adaptive response due to chronic BACE inhibition. Similar findings were recently reported on the clinical trial of atabecestat in early AD^45^. More recently, Biogen and Eisai discontinued two phase III clinical trials (Mission AD1 and AD2, in MCI and early AD patients, respectively) with BACE inhibitor elenbecestat due to unfavorable risk-benefit ratio. Our findings suggest that BACE1 inhibition to a lower extent than currently under investigation should be performed to preserve BACE1-mediated process of substrates involved in synaptic plasticity. The study of cKO mice in parallel with pharmacological BACE inhibition in animal models will facilitate the understanding of the mechanism(s) underlying the cognitive worsening observed in AD patients treated with BACE inhibitors, distinguish between BACE1-dependent phenotypes and off-target effects and finally improve drug dosing for future clinical applications.

## Materials and Methods

### Animals

BACE1^flox/flox^ mice (Supplementary Fig. 1a), kindly provided by Matthew Kennedy and Thomas Rosahl at Merck &Co. Inc. were crossed with RosaCreERT2^+/+^ mice (Jackson Laboratory, Stock No: 008463) to obtain mice homozygous for BACE1 floxed allele and hemizygous for the RosaCreERT2allele. BACE1^flox/flox^; RosaCreERT2^+/WT^ mice were used for all the experiments (Supplementary Fig. 1b). All mice generated were housed by standard conditions with ad libitum access to food and water. For Western blots, serial fractionation, Elisa and Real-Time quantitative PCR samples were collected as follow, mice were anesthetized with isoflurane and euthanized by vertebral dislocation. Cortex, hippocampus and cerebellum were dissected and snap frozen in liquid nitrogen. Samples were stored at −80 °C until use. All experimental procedures were approved by Tufts University Institutional Animal Care and Use Committees in accordance with US National Institutes of Health guidelines.

### Tamoxifen administration

Tamoxifen (Sigma) was dissolved in sunflower oil (Sigma) at a final concentration of 20 mg/ml. Animals were injected with a daily dose of 200 mg/kg per body weight of tamoxifen for 3 non-consecutive weeks (Fig. 1a). Mice were monitored and if adverse effects became apparent treatment was stopped.

### Western Blots and serial fractionation

Immunoblot and serial fractionation were performed as previously described^46^ with the following antibodies: rabbit monoclonal anti-BACE1 (1:1000; D10E5; Cell signaling technology); mouse monoclonal anti-APP (and APP CTFs) antibody (1:5000; C1/6.1; BioLegend); goat polyclonal anti-N-terminal CHL1 antibody (for CHL1-FL and CHL1-NTF) (1:1000; AF2147; R&D Systems); mouse monoclonal anti-GAPDH (1:10,000; MAP374; Millipore); mouse monoclonal anti-β-tubulin (1:10,000; JDR.3BR; Sigma); mouse monoclonal anti-calnexin (1:2000; 610523; BD biosciences); rabbit polyclonal anti-ADAM10 (1:1000;AB19026; Millipore); rabbit polyclonal anti-PS1 AB14 (1:1000)^47^ and rat monoclonal anti-SEZ6 (1:250)^25^, rat monoclonal anti-APPsβ (1:40)^48^ and HRP-conjugated secondary antibodies visualized by ECL (GE Healthcare). Chemiluminescent signal was captured on an LAS4000 Fuji Imager. Densitometry analysis was performed using Quantity One software (Bio-Rad Laboratories).

### Real-time quantitative PCR

Total RNA was extracted using RNAeasy Mini kit (Qiagen) subjected to reverse transcription with QuantiTect Reverse Transcription kit (Qiagen), according to manufacturer’s instructions. RNA was analysed with the Agilent 2100 Bioanalyzer (Agilent Technologies). Reverse transcription reaction was amplified using the SYBR Green PCR Master Mix kit (Life Technologies) in a MX3000p qPCR System (Agilent Technologies). Primers were used were: BACE1 (Primer Bank ID: 225543121c1) and GAPDH (Primer Bank ID: 6679937a1). Relative expression levels were determined according to the ΔΔCT method.

### Aβx-40 quantification

Homogenates in RIPA buffer were quantified by ELISA Kit Wako (Catalog #294–62501, Fujifilm Wako Pure Chemicals Corporation) or Meso Scale Discovery platform (V-PLEX Plus Aβ peptide 4G8 Kit, Meso Scale Diagnostics) according to manufacturer’s instruction.

### Histology

Mice were anesthetized with isoflurane, perfused intracardially with ice-cold PBS followed by 50 ml of ice-cold 4% PFA. Brains were post fixed over night at 4 °C in 4% PFA, washed in PBS and incubated in cryoprotectant solution (30% sucrose, 1% Polyvinyl-pyrrolidone-40, 0.05 M phosphate buffer, 30% ethylene glycol) for at least 48 hours. Coronal sections 40 µm thick were cut using a sliding microtome and stored in cryoprotectant solution. 3 to 4 sections per mouse (VEH n = 8; TAM n = 7) at anterior/posterior coordinate spanning from −1.5 to −2.5 from Bregma were selected for IPB analysis. Sections were washed in PBS, then blocked in 5% BSA +0.25% of Triton-X 100 and incubated with the following antibodies: anti-synaptoporin (102003, 1:500, Synaptic Systems) anti-BACE1 (D10E5, 1:1000, Cell signaling technology), anti-rabbit Alexa Fluor 488 (Life Technologies). Fluorescent images were acquired on BZ-X700 all-in-one fluorescence microscope (Keyence). Images from SPO staining were used for IPB length quantification with Fiji software (Image J)^49^. IPB length was normalized on MB + slu length.

For immunohistochemistry sections were washed with peroxidase blocking solution (3%H2O2, 1% MetOH in PBS), blocked with Superblock (Pierce) and incubated with the primary antibody (anti-BACE1 EPR3956, 1:250, Abcam). The next day sections were washed with PBS, incubated with anti-rabbit biotinylated secondary antibody (1:500, Vector Labs, Burlingame), then incubated with ABC complex (Vector Labs, Burlingame) and developed with DAB (Sigma).

### G-ratio quantification in sciatic nerve

Sciatic nerves were perfused with 4% PFA/PBS, immersion fixed in 4% glutaraldehyde/PBS overnight, embedded in EPON resin and examined using a JEOL JEM-1011 transmission electron microscope with AMTv601 software (Advanced Microscopy Techniques). Tissue processing and TEM were conducted in the laboratory of Dr. Marian DiFiglia by Erin Jones (Massachusetts General Hospital, Department of Neurology, Charlestown, MA). G-ratio quantification was performed with Fiji software (Image J)^49^.

### Electrophysiological recording

Coronal hippocampal slices were prepared from 14 month-old BACE1^flox/flox^;RosaCreERT2^+/WT^ as described previously^50^. Schaffer collaterals were electrically stimulated at 0.05 Hz with a concentric tungsten electrode placed in the stratum radiatum. Evoked field excitatory postsynaptic potentials (fEPSPs) were recorded using a glass electrode (2–4 MΩ) filled with aCSF and placed in the stratum radiatum. For characterization of basal synaptic transmission, fEPSPs, generated by gradually increasing stimulus intensity, were recorded. For LTP experiments, after a stable fEPSP had been recorded for at least 10 min (using a response about 50% of the maximum), LTP was induced with three trains of 100 Hz for 1 s with an intertrain interval of 20 s. Data were collected and analysed using pCLAMP 9 software (Molecular Devices).

### Behaviour

All behavioural testing was conducted in the Tufts University Center for Neuroscience Research (CNR) Circuits and Behavior Core Facility. A total number of 21 TAM-treated mice (14 males, 7 females) and 19 VEH-treated mice (13 males, 6 females) were subjected to a battery of behavioural tests at two different time points [4–5 month-old (young) and 12–13 month-old (aged)]. The tests were performed from the least stressful test to the more stressful one in the following order: open field, light/dark transition, Y-maze, pre-pulse inhibition (PPI) and contextual fear conditioning. Chambers were thoroughly cleaned with 70% ETOH between test subjects. Open field, light/dark transitions and contextual fear conditioning were performed as previously described^51^.

#### Y-maze

Mice were placed in the starting arm and given 8 min to freely explore and recorded using EthoVision^®^ XT10 (Noldus Information Technology). The correct sequential triad (e.g., ABC) entry into all three arms of the y-maze was classified as a ‘correct alternation event’ whereas an incorrect sequential triad (e.g., ABA) was classified as ‘incorrect alternation event’. All data were reported as %alternation.

#### Pre-pulse inhibition (PPI)

Animals were tested for PPI using the Startle Monitor System (Kinder Scientific). The startle chamber (inner dimensions: 27 L × 17 W cm × 28 H cm) consisted of a speaker (diameter 7 cm) mounted on the top the chamber pointing down and a piezoelectric sensing platform on the floor. During testing, animals were placed in an adjustable holder (9 L × 4 W × 3.5 H cm) positioned atop the sensing platform, providing only limited restraint while prohibiting ambulation. Animals were allowed a 5 min acclimation period prior to the onset of acoustic stimuli. A continuous 65 dBA broadband (“white”) background noise was present during the acclimation period and throughout the experiment. Two trial types were used in the experiment: basic startle stimulus of 40 mS, 120 dBA broad band noise pulse, and a pre-pulse stimuli consisting of several 40 mS, 65, 69, 73, 77, and 81 dBA pre-pulses that preceded the basic startle stimulus by 120 mS. No stimulus trials were also included. These two stimulus trial types are referred to herein as pulse and pre-pulse trials, respectively. Each animal received 72 stimuli presented in pseudorandom order with a pseudorandom interstimulus interval (range 7–23 sec). The first and last 6 trials consisted of a 120 dBA pulse stimuli to provide a baseline startle response. Whole body startle responses were recorded in Newtons. The percent of PPI expressed within each test session was calculated as follows: [100 − (mean pre-pulse response/mean pulse response) × 100)].

### Statistical analysis

Data from the biochemical and immunohistochemistry studies were analysed by two-tailed Student’s t-tests. Data from G-ratio quantification were analysed with Mann-Whitney test (to detect difference between medians) and Kolmogorov-Smirnov test (to check for significant difference in the distribution). Input output curve was analysed by 2-way ANOVA, EPSP vs fiber volley curves were analysed by linear regression and LTP was analysed by Mann–Whitney U-test.

Behavioural studies were analysed with 3-way repeated measure ANOVAs (time, treatment and sex as independent variables). When no sex-treatment interactions were detected, male and female mice were pooled and 2-way repeated measures ANOVAs were performed (time and treatment as independent variables). A sex difference was observed for the distance travelled in the open field, in this case male and female mice were analysed separately using a 2-way ANOVA with time and treatment as independent variables. Statistical analysis was performed with GraphPad PRISM software, v. 7 (GraphPad Software).

## Supplementary information

Supplementary information

## Data Availability

The datasets generated during and analysed during the current study are available from the corresponding author on reasonable request.
