# Supplementary material for: BACE1 partial deletion induces synaptic plasticity deficit in adult mice
Source: Sci Rep. 2019 Dec 27;9:19877. doi: 10.1038/s41598-019-56329-7 (PMC6934620; doi:10.1038/s41598-019-56329-7)

BACE1 partial deletion induces synaptic plasticity deficit in adult mice

Sylvia Lombardo[1,2], Martina Chiacchiaretta[2], Andrew Tarr[3], WonHee Kim[1,2], Tingyi Cao[1,2], Griffin Sigal[1,2], Thomas W. Rosahl[4], Weiming Xia[5,6], Philip G. Haydon[2], Matthew E. Kennedy[7], and Giuseppina Tesco[1,2].

[1]Alzheimer's Disease Research Laboratory, Department of Neuroscience, Tufts University School of Medicine, Boston, Massachusetts 02111, USA.

[2]Department of Neuroscience, Tufts University School of Medicine, Boston, Massachusetts 02111, USA.

[3]Circuits and Behaviour Core, Center for Neuroscience Research, Tufts University School of Medicine, Boston, Massachusetts 02111, USA.

[4]External In Vivo Pharmacology, Merck & Co. Inc., Kenilworth, NJ 07033, USA.

[5]Geriatric Research, Education and Clinic Center, Bedford Veterans Affairs Medical Center, Bedford, MA 01730, USA.

[6]Department of Pharmacology and Experimental Therapeutics, Boston University School of Medicine, Boston, MA 02118, USA.

[7]Department of Neuroscience, Merck & Co. Inc, Boston, MA 02115, USA.

Corresponding author: Giuseppina Tesco. Email: giuseppina.tesco@tufts.edu; Alzheimer's Disease Research Laboratory, Department of Neuroscience, Tufts University School of Medicine, Boston, Massachusetts 02111 Tel. 617 636 4050; Fax: 617 636 2413.

Supplementary Information

Table S1

Repeated Measure ANOVA (3 and 2 way interaction)						
**Y Maze 3WA**	**Interaction (3WA)**		**Treatment (vehicle, tamoxifen)**		**Sex (male, female)**	
Percentage of alternations	F (1, 36) = 0.067	P = 0.797	F (1, 36) = 0.101	P = 0.752	F (1, 36) = 0.055	P = 0.815
**Y Maze 2WA**	**Interaction (2WA)**		**Treatment (vehicle, tamoxifen)**		**Time (4-5, 12-13 month-old)**	
Percentage of alternations	F (1, 38) = 0.063	P = 0.801	F (1, 38) = 1.113	P = 0.298	F (1, 38) = 0.720	P = 0.401
**Fear Conditioning 3WA**	**Interaction (3WA)**		**Treatment (vehicle, tamoxifen)**		**Sex (male, female)**	
Freeze percentage	F (1, 36) = 0.020	P = 0.888	F (1, 36) = 0.514	P = 0.478	F (1, 36) = 0.002	P = 0.961
**Fear Conditioning 2WA**	**Interaction (2WA)**		**Treatment (vehicle, tamoxifen)**		**Time (4-5, 12-13 month-old)**	
Freeze percentage	F (1, 38) = 0.709	P = 0.404	F (1, 38) = 0.064	P = 0.800	F (1, 38) = 0.801	P = 0.376
**Open Field 3WA**	**Interaction (3WA)**		**Treatment (vehicle, tamoxifen)**		**Sex (male, female)**	
Total distance	F (1, 36) = 0.677	P = 0.416	F (1, 36) = 0.478	P = 0.494	F (1, 36) = 4.794	P = 0.035
Time in center	F (1, 36) = 1.668	P = 0.204	F (1, 36) = 0.020	P = 0.889	F (1, 36) = 1.911	P = 0.175
**Open Field 2WA**	**Interaction (2WA)**		**Treatment (vehicle, tamoxifen)**		**Time (4-5, 12-13 month-old)**	
Total distance (male)	F (1, 25) = 0.011	P = 0.915	F (1, 25) = 0.093	P = 0.762	F (1, 25) = 14.71	P < 0.001
Total distance (female)	F (1, 11) = 0.172	P = 0.685	F (1, 11) = 0.064	P = 0.804	F (1, 11) = 0.599	P = 0.455
Time in center	F (1, 38) = 352	P = 0.556	F (1, 38) = 0.182	P = 0.666	F (1, 38) = 1.604	P = 0.213
**Light Dark transitions 3WA**	**Interaction (3WA)**		**Treatment (vehicle, tamoxifen)**		**Sex (male, female)**	
Time light zone	F (1, 36) = 0.351	P = 0.557	F (1, 36) = 0.174	P = 0.679	F (1, 36) = 2.820	P = 0.101
**Light Dark transitions 2WA**	**Interaction (2WA)**		**Treatment (vehicle, tamoxifen)**		**Time (4-5, 12-13 month-old)**	
Time light zone	F (1, 38) = 0.035	P = 0.850	F (1, 38) = 1.371	P = 0.248	F (1, 38) = 0.777	P = 0.383

Repeated Measure ANOVA (3 and 2 way interaction)						
**PPI (4-5 month-old) 3WA**	**Interaction (3WA)**		**Treatment (vehicle, tamoxifen)**		**Sex (male, female)**	
Pre pulse inhibition	F (3, 117) = 0.895	P = 0.446	F (3, 117) = 1.482	P = 0.223	F (3, 117) = 0.219	P = 0.882
**PPI (4-5 month-old) 2WA**	**Interaction (2WA)**		**Treatment (vehicle, tamoxifen)**		**dB**	
Pre pulse inhibition	F (3, 123) = 1.865	P = 0.138	F (1, 41) = 3.206	P = 0.080	F (3, 123) = 73.65	P <0.0001
**PPI (12-13 month-old) 3WA**	**Interaction (3WA)**		**Treatment (vehicle, tamoxifen)**		**Sex (male, female)**	
Pre pulse inhibition	F (3, 108) = 0.392	P = 0.759	F (3, 108) = 0.827	P = 0.481	F (3, 108) = 0.750	P = 0.524
**PPI (12-13 month-old) 2WA**	**Interaction (2WA)**		**Treatment (vehicle, tamoxifen)**		**dB**	
Pre pulse inhibition	F (3, 114) = 1.085	P = 0.358	F (1, 38) = 1.641	P = 0.207	F (3, 114) = 135.2	P <0.0001

Table S1. Summary of 3-way and 2-way ANOVAs. For 3-way ANOVA we reported: 3-way interaction, time and treatment interaction and sex and time interaction. 3WA: 3-way ANOVA; 2WA: 2-way ANOVA.

Figure S1

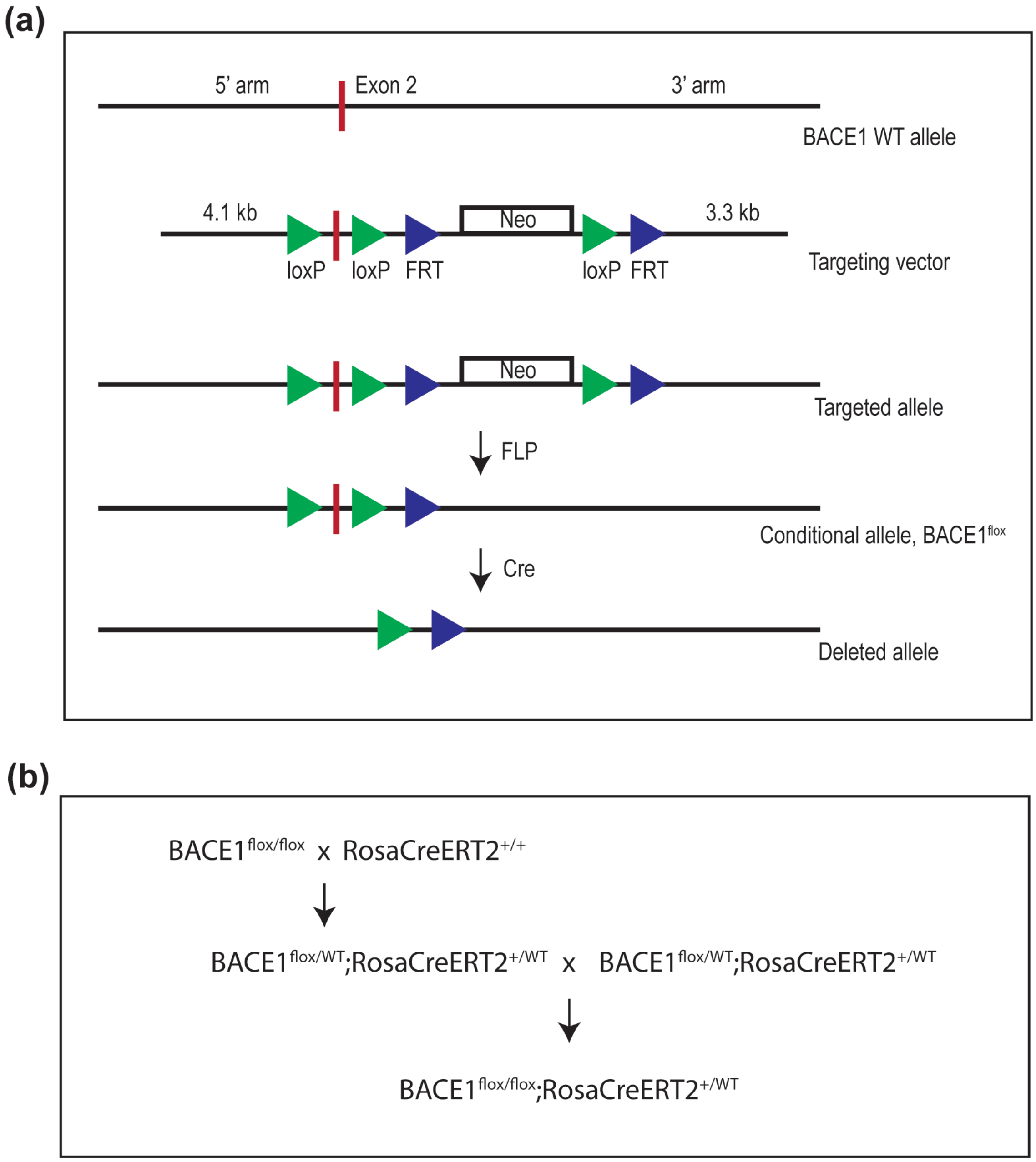

Figure S1. Generation of BACE1$^{flox/flox}$ line and breeding strategy used. **a)** Schematic representation of Exon2 targeting strategy, showing BACE1 genomic locus containing Exon2, targeting vector (containing the Exon2 flanked by loxP sequences) used for site specific recombination, targeted allele obtained after recombination, conditional allele obtained after removal of Neo selection cassette and finally, the resulting deleted allele obtained after TAM-

induced recombination and Exon2 excision. **b)** Breeding strategy used to obtain mice homozygous for the BACE1$^{flox/flox}$ allele and hemizygous for RosaCreERT2 allele. All mice used for the experiments described in Fig.1-7 were of the BACE1$^{flox/flox}$;RosaCreERT2$^{+/WT}$ genotype.

Figure S2

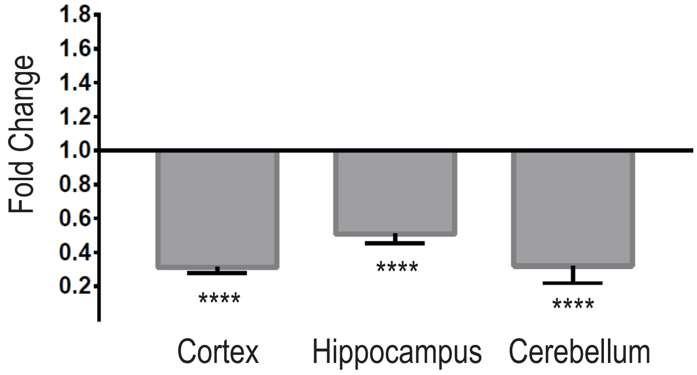

Figure S2. Quantitative real-time PCR (Q-PCR) of BACE1 gene. BACE1 mRNA levels were significantly decreased in cortex, hippocampus and cerebellum of TAM-treated mice collected at 4-5 months of age. Data were expressed as Fold Change Mean ± Confidence Interval (CI) at 95% (VEH n=7; TAM n=9), **** $p < 0.0001$, Student’s t test.

Figure S3

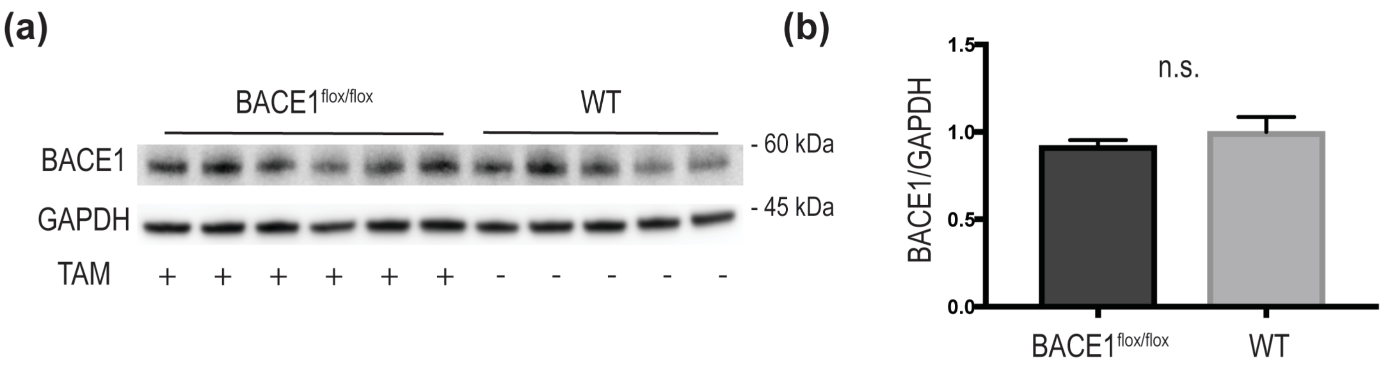

Figure S3. BACE1 levels in BACE1$^{flox/flox}$ TAM-treated mice. **a)** Representative blot of BACE1$^{flox/flox}$ TAM-treated and untreated wild-type mice (WT). **b)** Quantification of blots showed no change in BACE1 protein levels in BACE1$^{flox/flox}$ compared to WT. BACE1 (D10E5) was normalized to GAPDH (MAB374) (BACE1$^{flox/flox}$ n=6; WT n=5). Results were plotted as Mean ± SEM, n.s.= not significant, Student's t test.

Figure S4

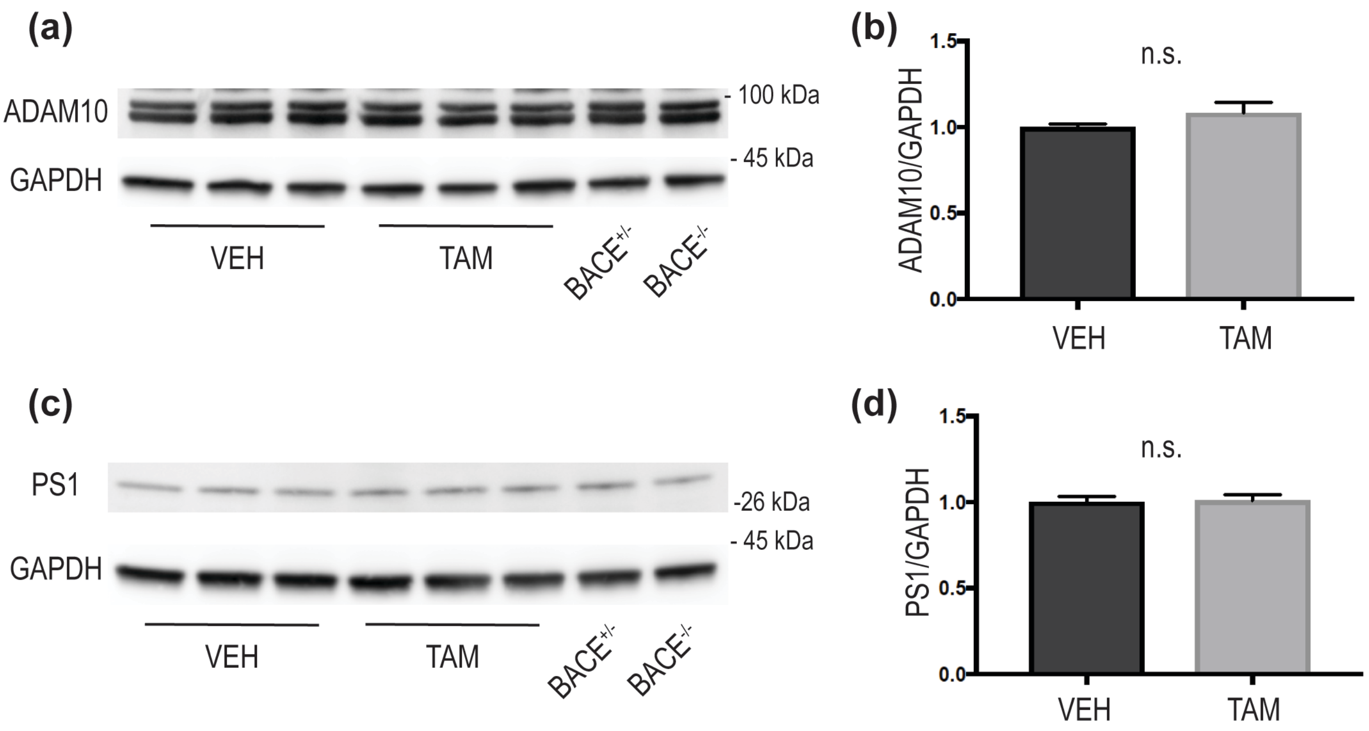

Figure S4. ADAM10 and PS1 protein levels in young TAM-treated mice. Representative blots of cortex homogenates from TAM- or VEH-treated mice for **a)** ADAM10 (AB19026) and **c)** PS1 (AB14), showing no changes in the levels of these proteins in mice TAM-treated compared to controls **(b and d)**. ADAM10 and PS1 were normalized to GAPDH (MAB374) (VEH n=8; TAM n=8). Results were plotted as Mean ± SEM, n.s.= not significant, Student’s t test.

Figure S5

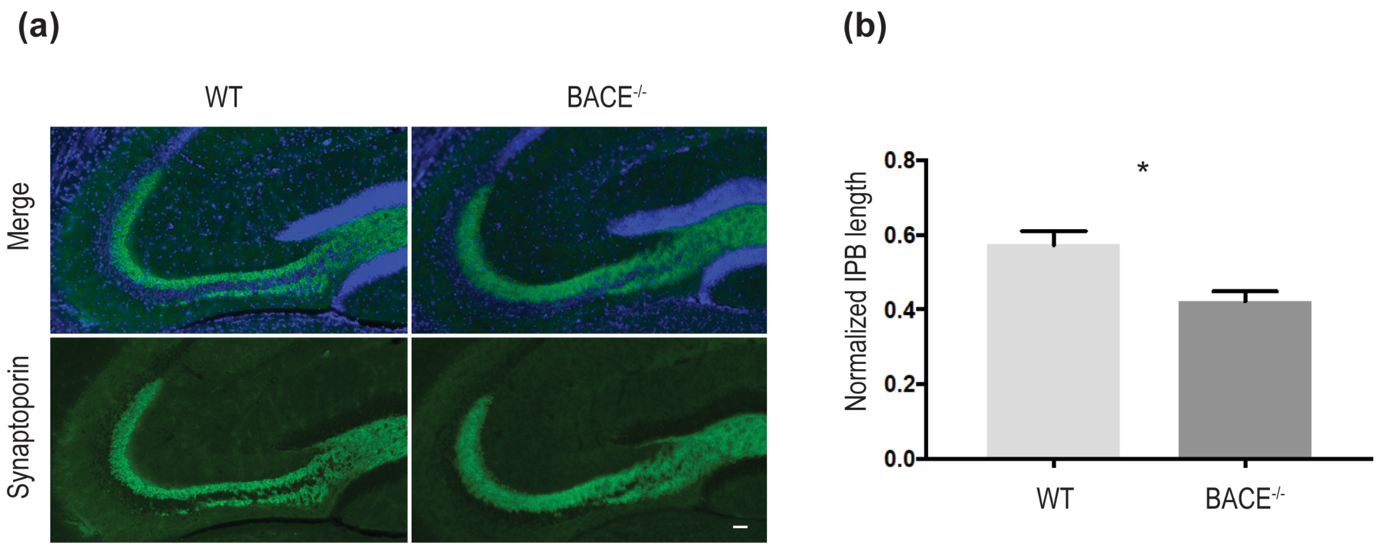

Figure S5. Impaired mossy fiber IPB morphology in BACE$^{-/-}$ mice **a)** Representative coronal sections from WT (left panel) and BACE$^{-/-}$ (right panel) from 12-13 month-old mice stained with anti-synaptoporin antibody (green) and DAPI (blue). **b)** Quantification showed that IPB length in BACE$^{-/-}$ is significantly shorter compared to WT mice. IPB length (μm) was normalized on the length of the CA3 stratum lucidum (WT n=5; BACE1$^{-/-}$ n=5). Scale bar 100 μm. Results were plotted as Mean ± SEM, * $p < 0.05$, Student's t test.

Uncropped Blots

Uncropped blot Figure 1a

BACE1

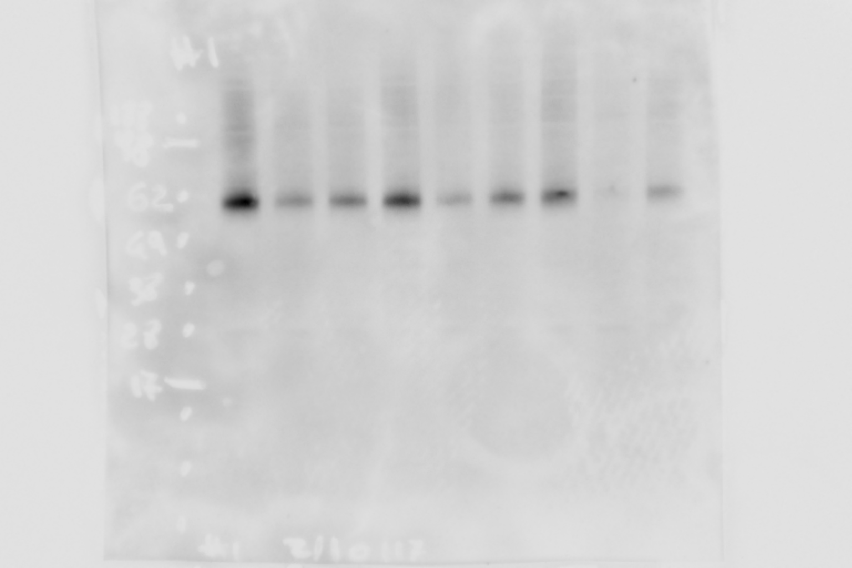

GAPDH

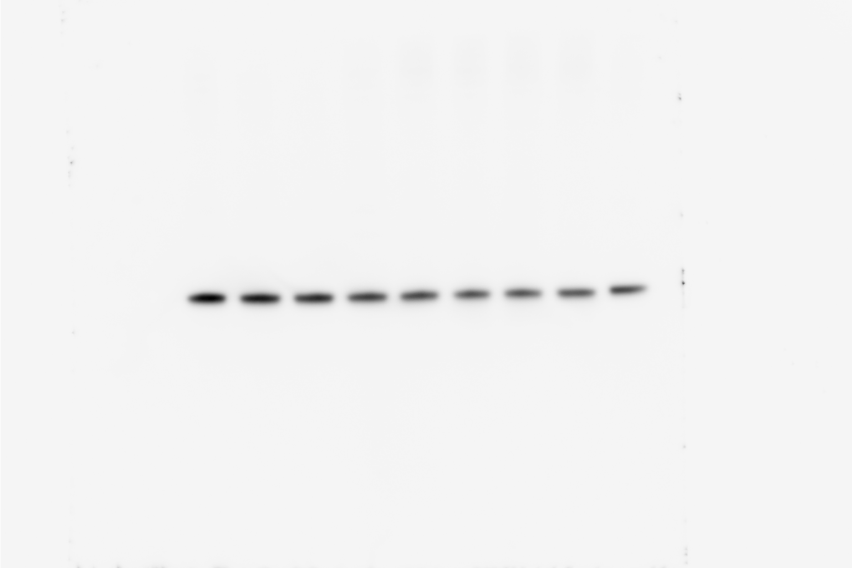

Uncropped blot Figure 1b

BACE1

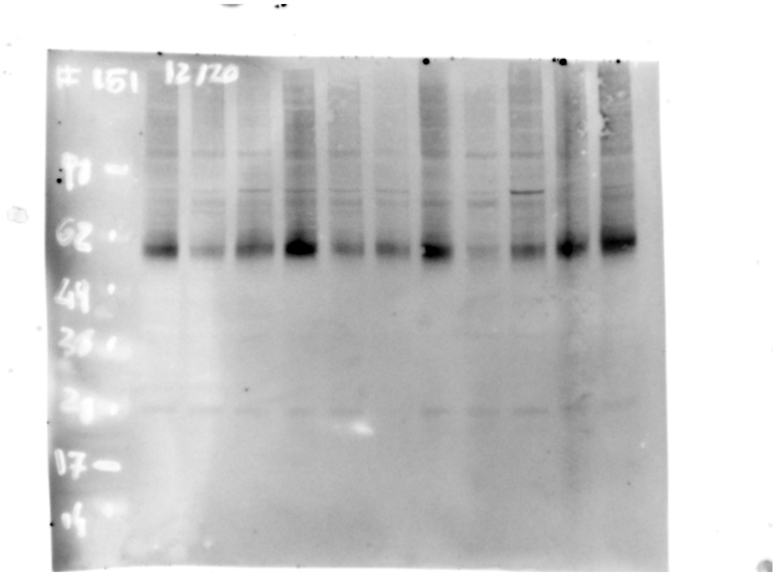

GAPDH

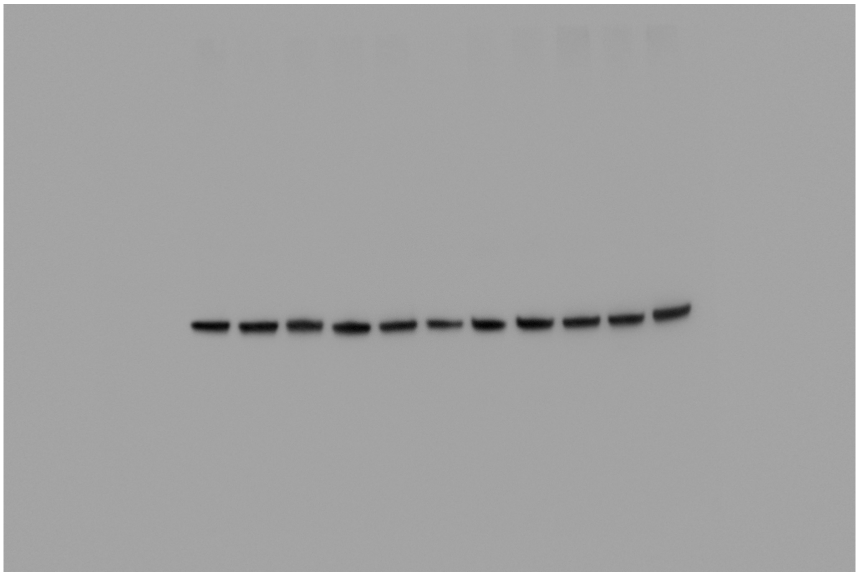

Uncropped blot Figure 2a

APP

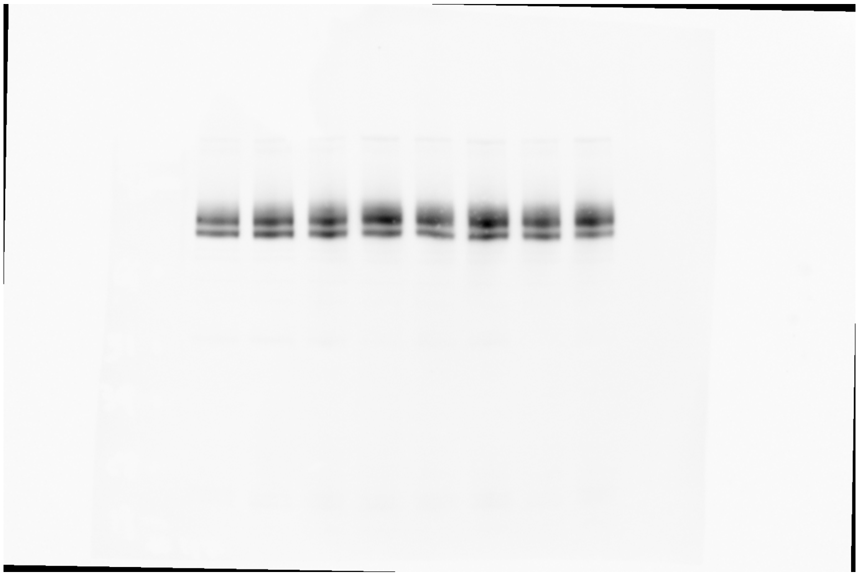

GAPDH (probed GAPDH on previous gel on which APP and CHL1 were detected)

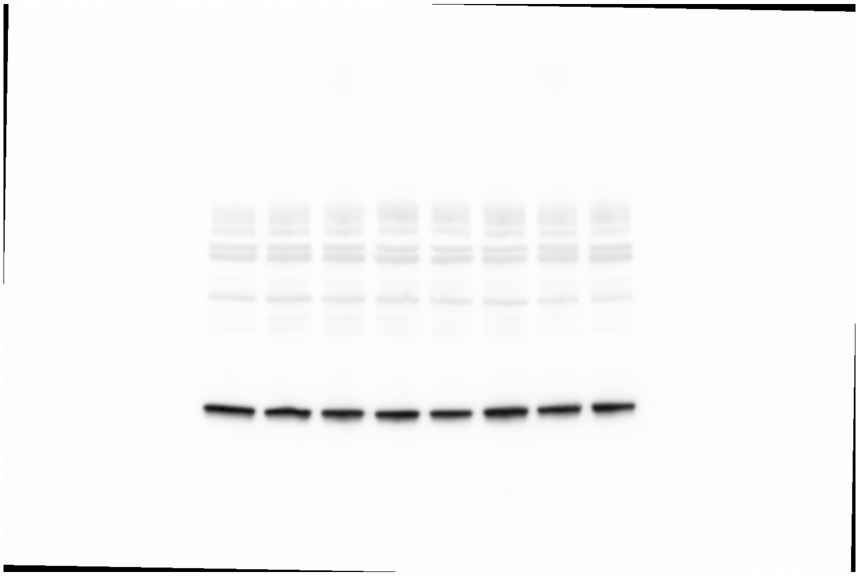

Uncropped blot Figure 2b

APP CTFs

Top of blot was covered to avoid saturation from APP-full length fragment

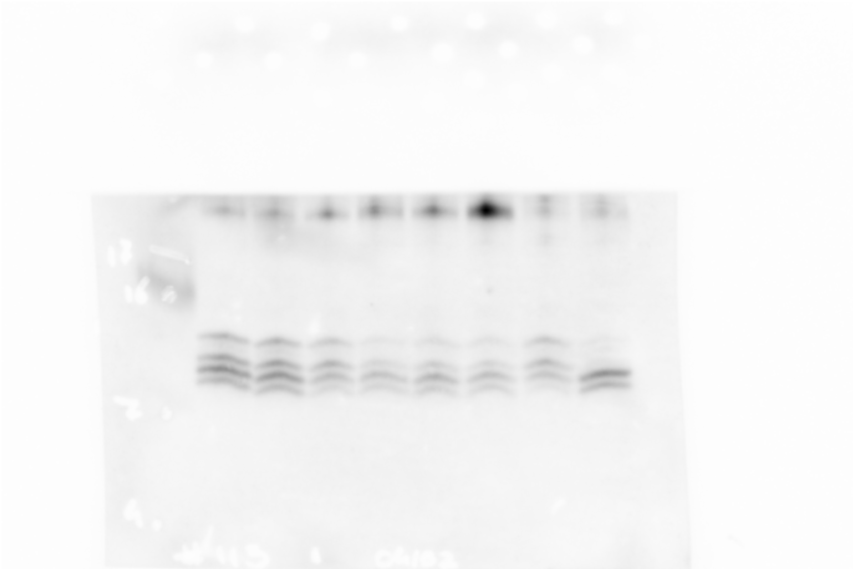

GAPDH

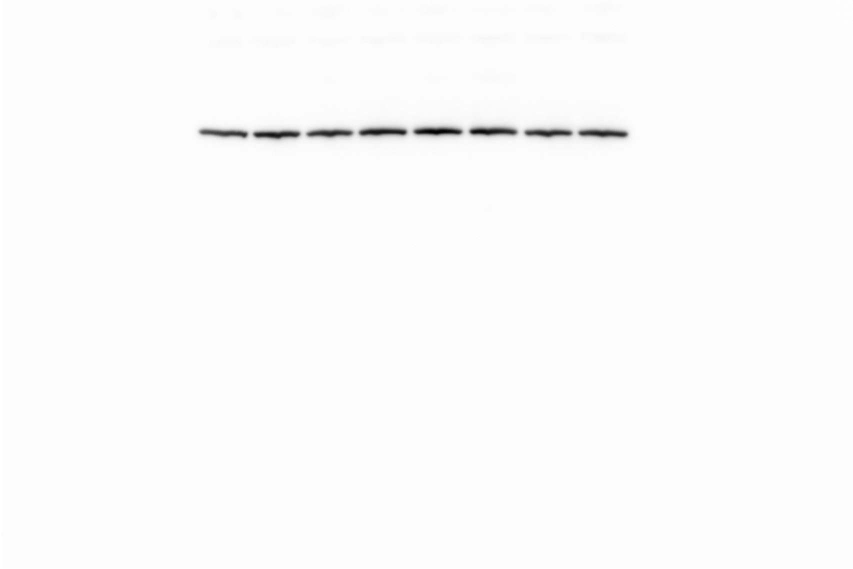

Uncropped blot Figure 2c

CHL1 (probed CHL1 on previous gel on which APP was detected)

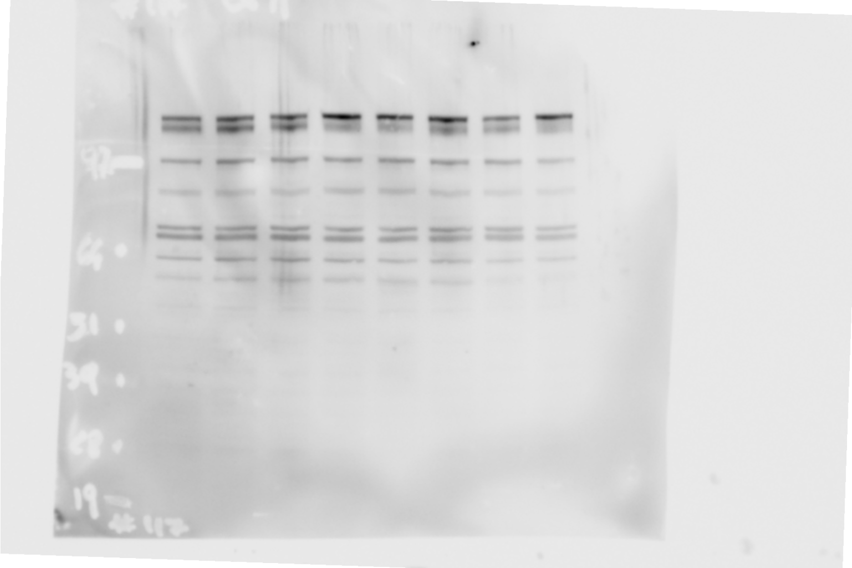

β-tubulin

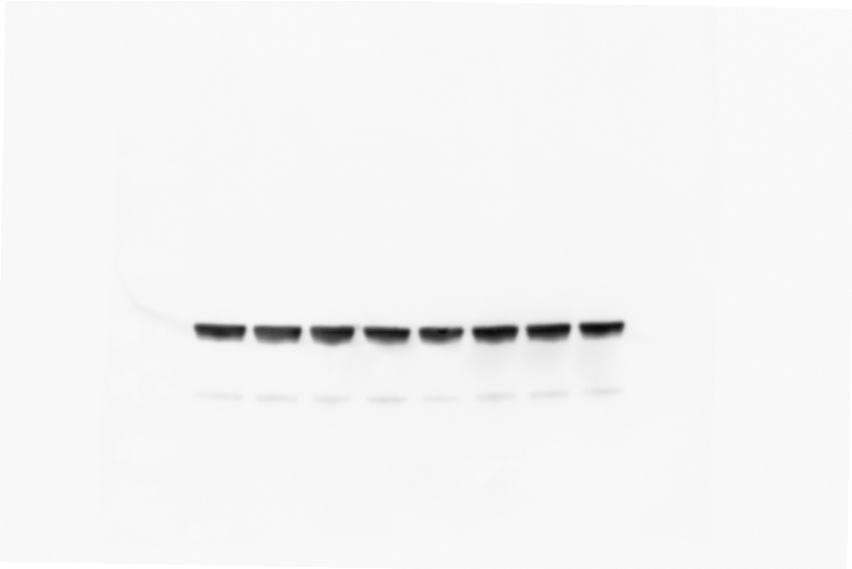

Uncropped blot from Figure 3a

APP

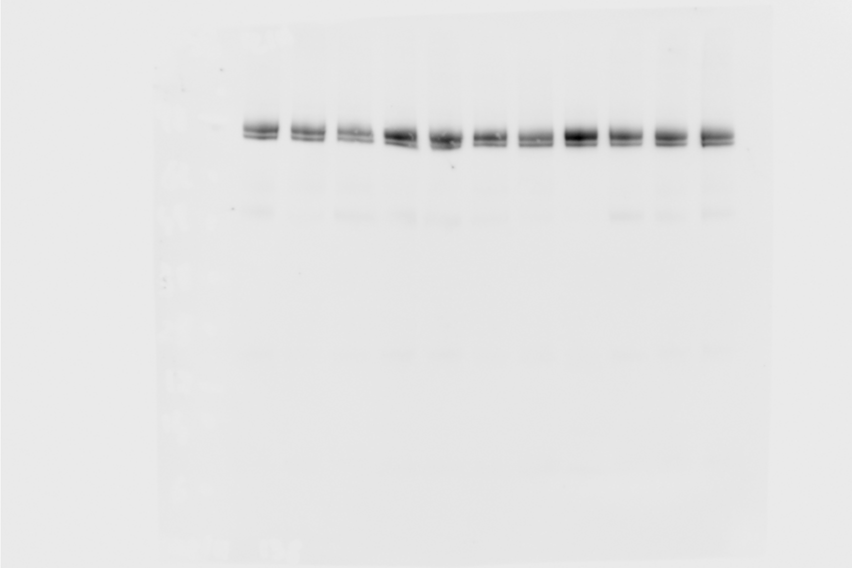

GAPDH

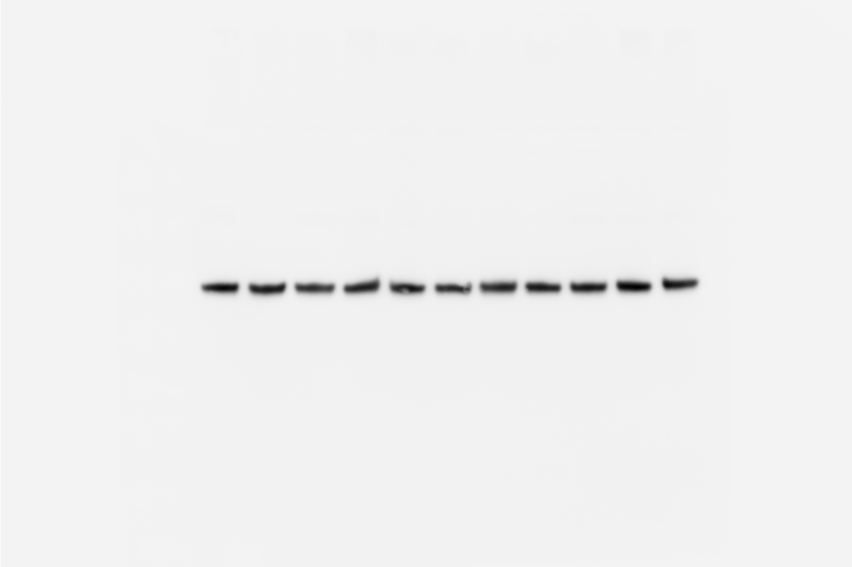

Uncropped blot from Figure 3b

APP CTFs

Top of blot was covered to avoid saturation from APP-full length fragment

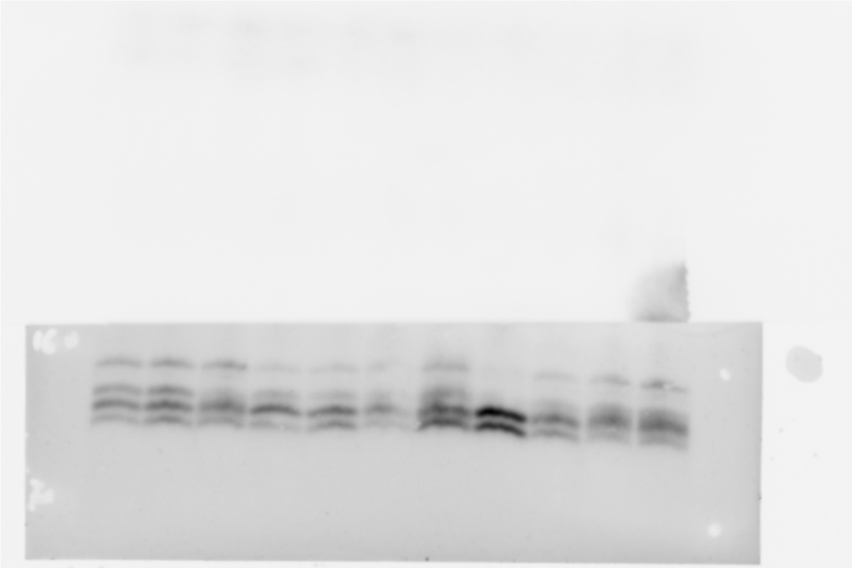

GAPDH

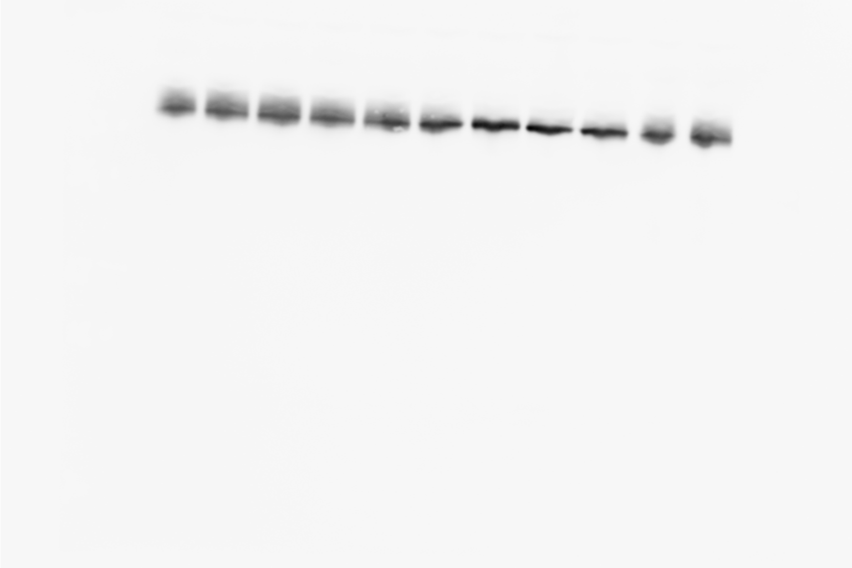

Uncropped blot from Figure 3c

CHL1

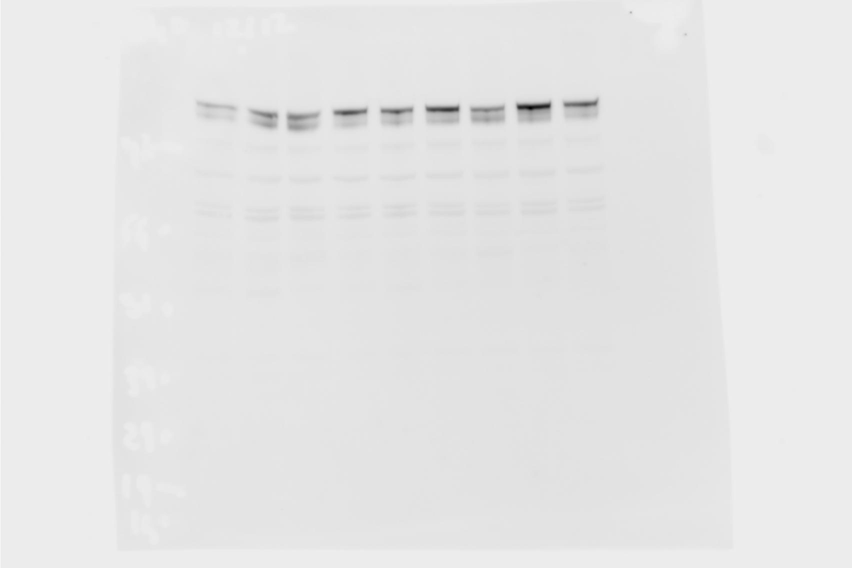

β-tubulin

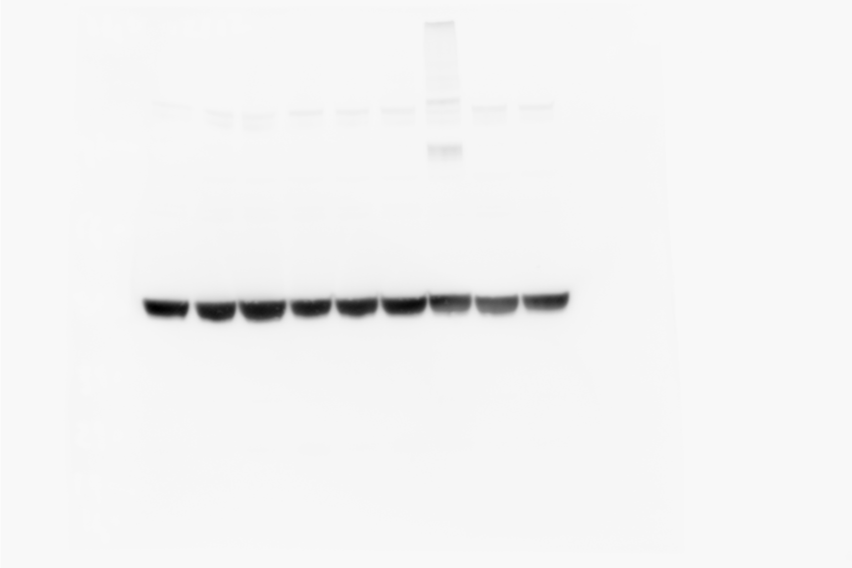

Uncropped blot from figure 5b

APP-FL

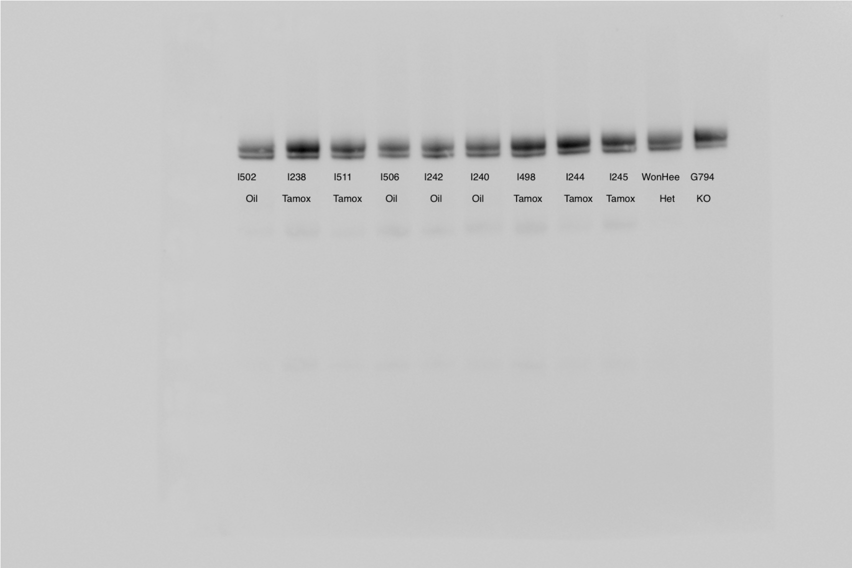

GAPDH

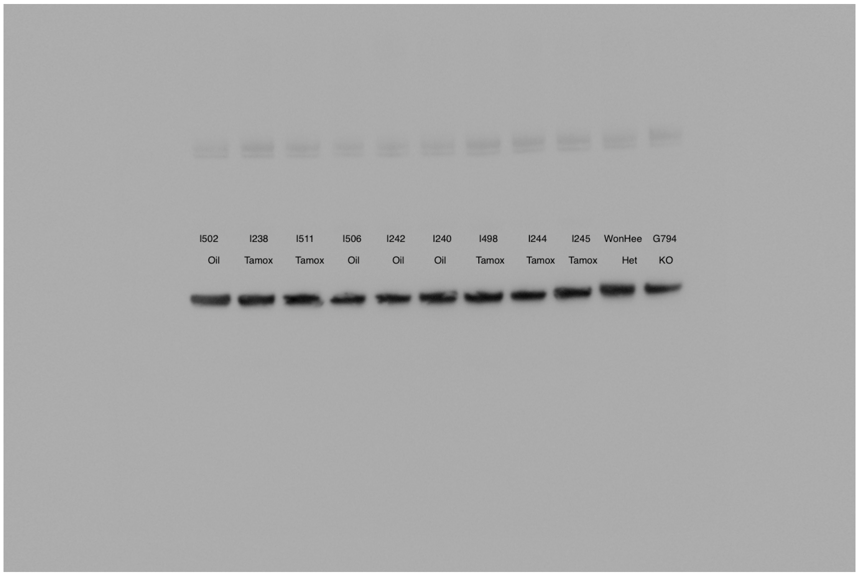

APP CTFs

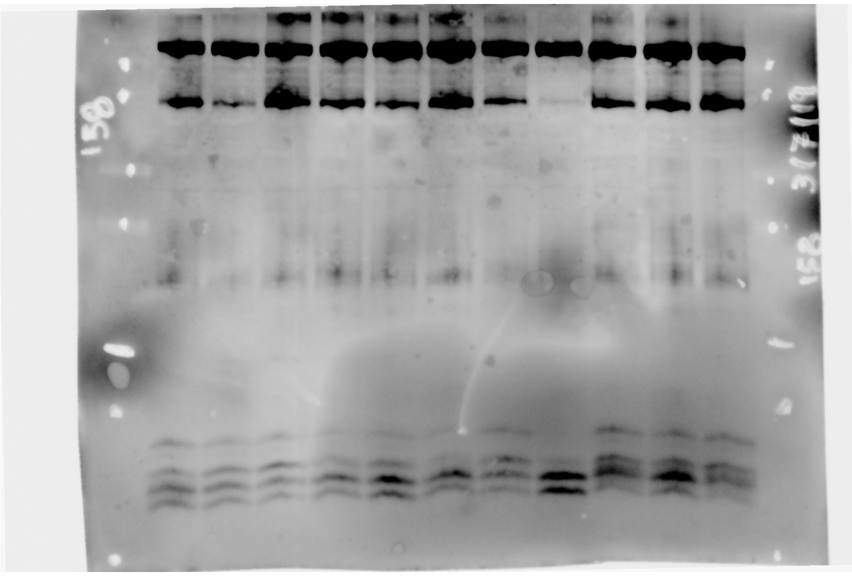

GAPDH

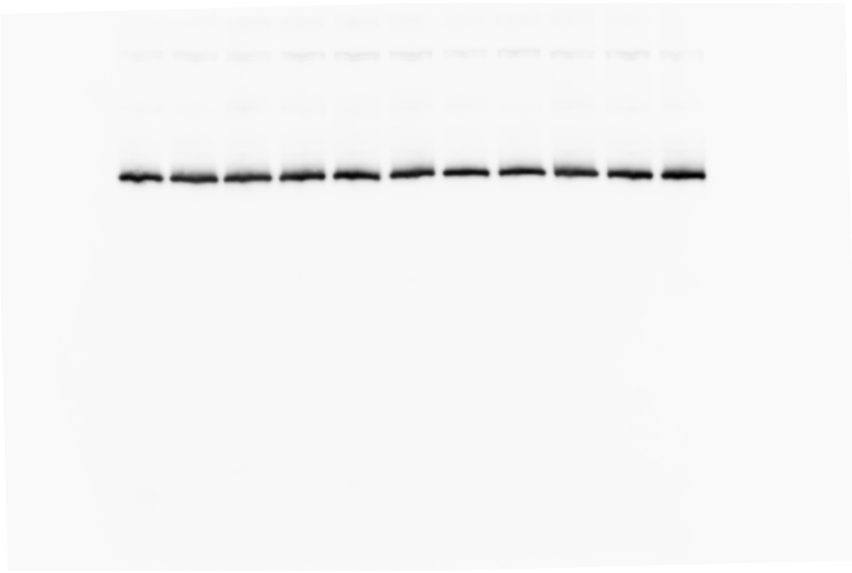

Uncropped blots Figure 5e

SEZ6-FL (top of the membrane was cover to avoid CHL1 signal, see blot below)

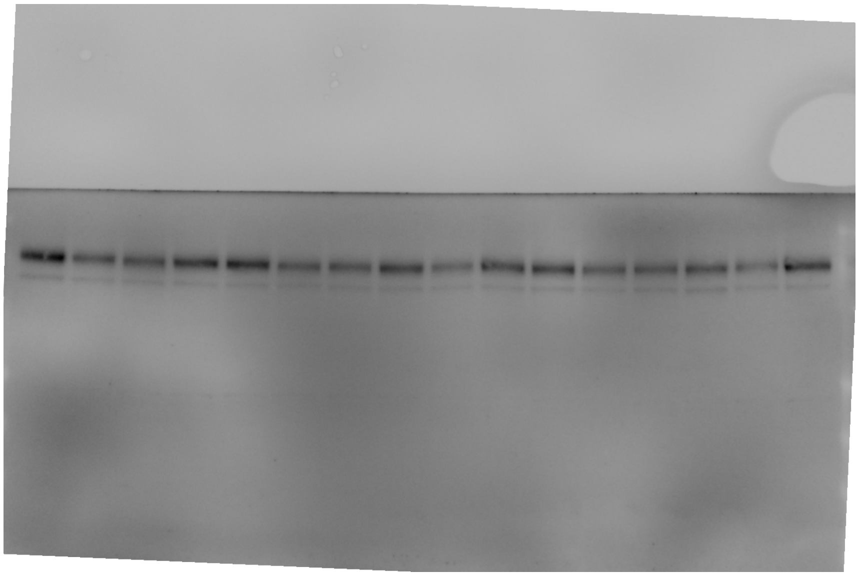

CHL1-FL

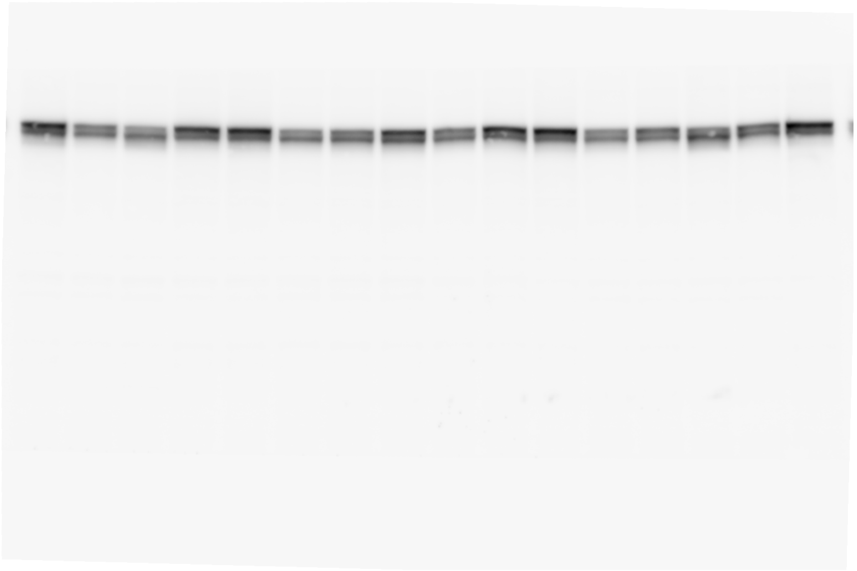

Calnexin (bottom of the membrane was cut)

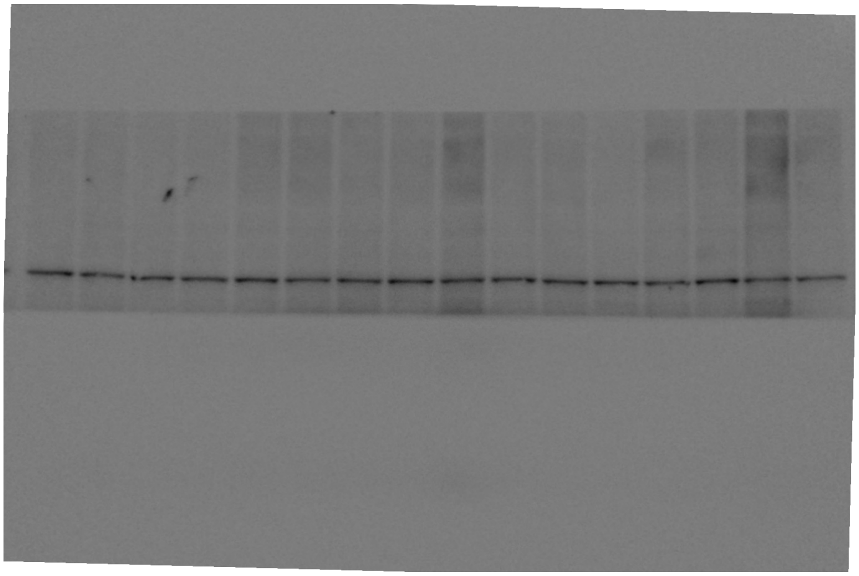

sAPPβ

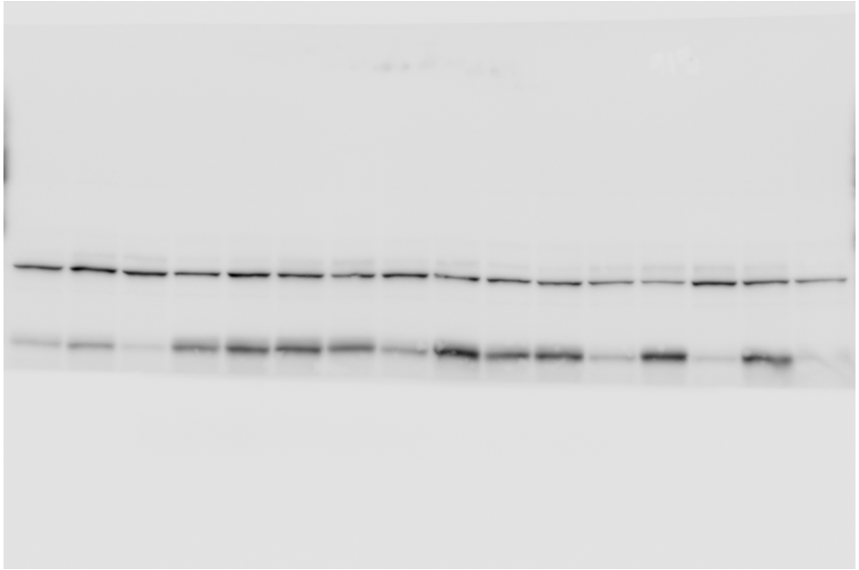

SEZ6-NTF

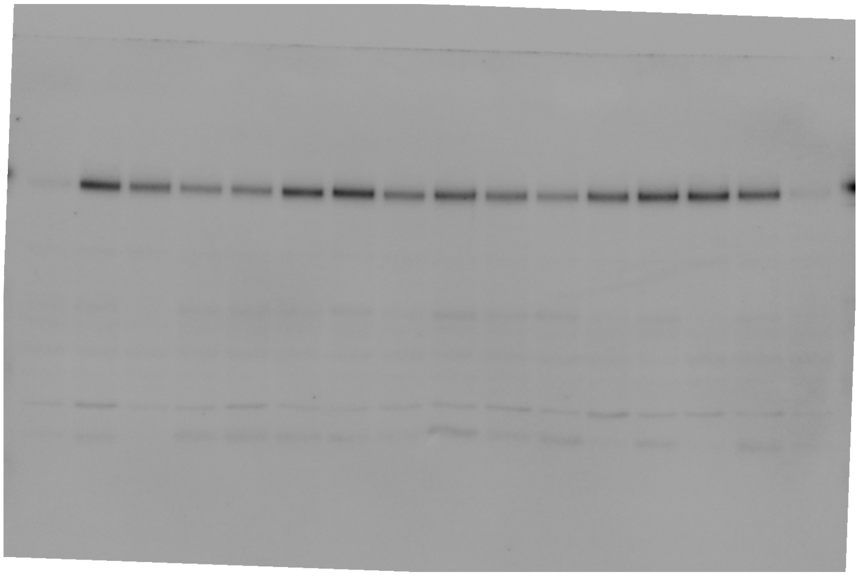

CHL1 NTF

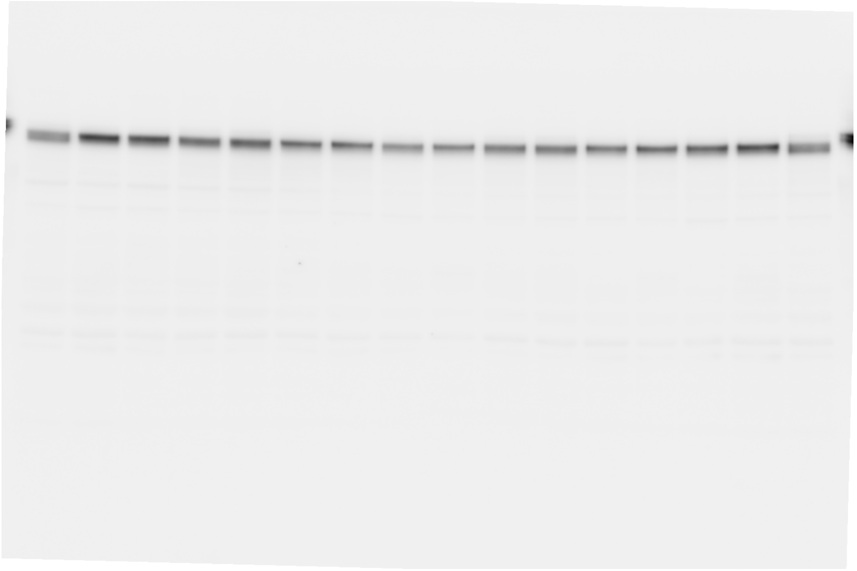

GAPDH

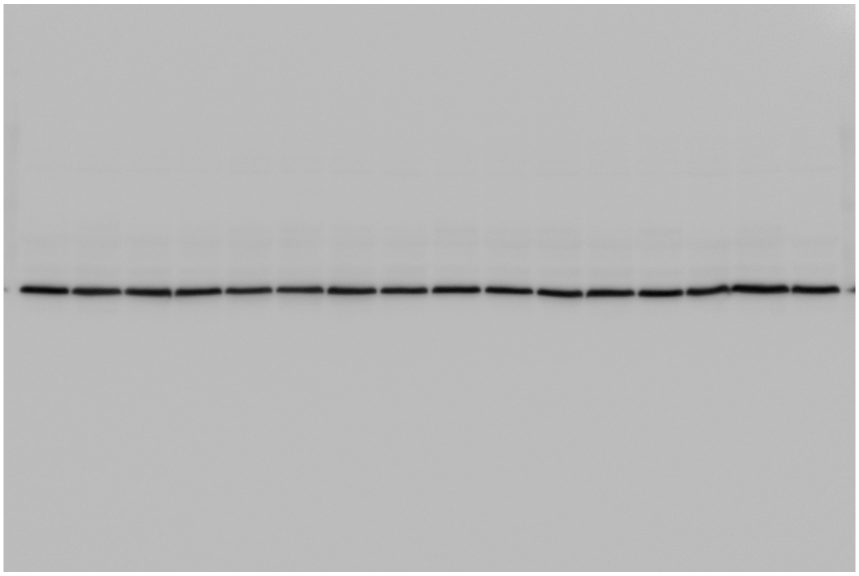

Uncropped blots Supplementary Figure 3a

BACE1

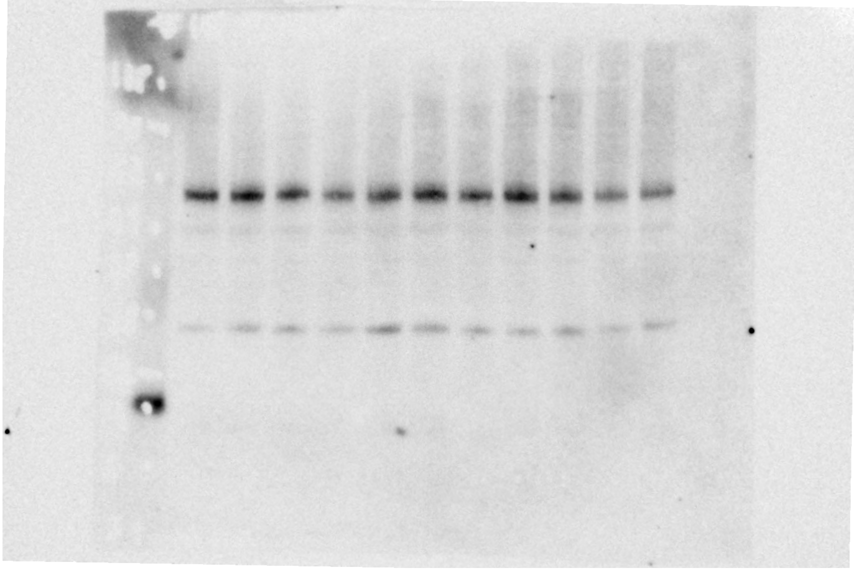

GAPDH

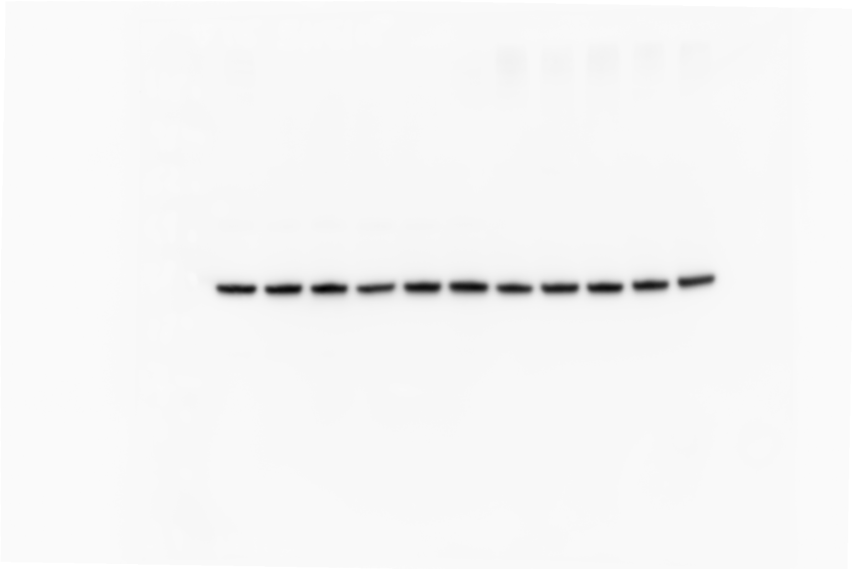

Uncropped blots Supplementary Figure 4a

ADAM10

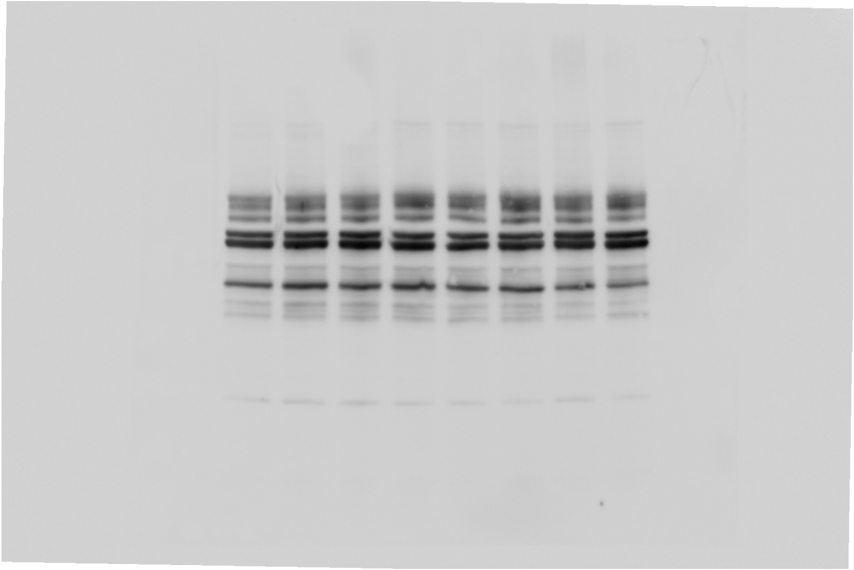

GAPDH

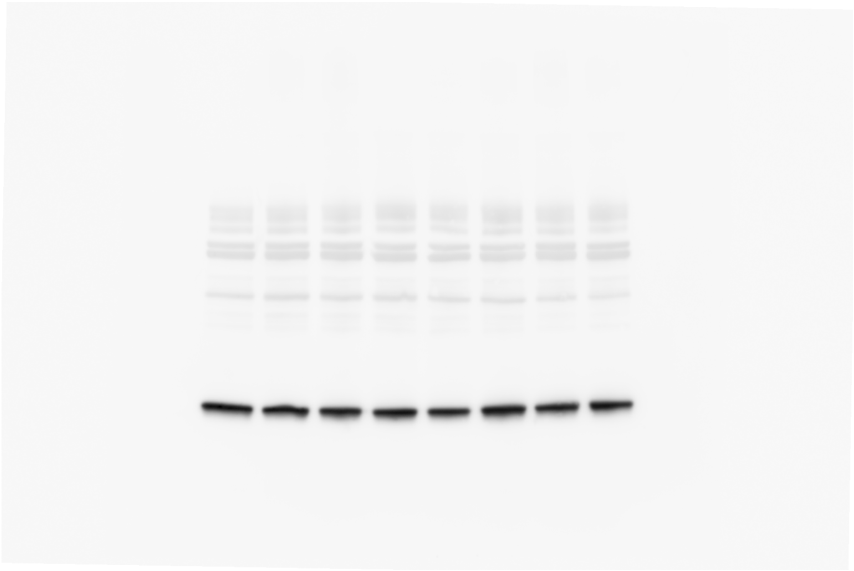

Uncropped blots Supplementary Figure 4b

PS1

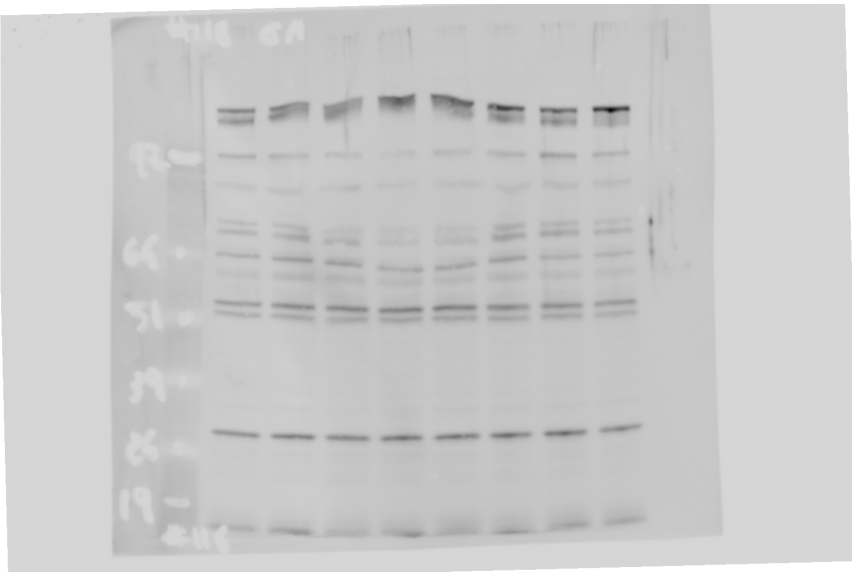

GAPDH

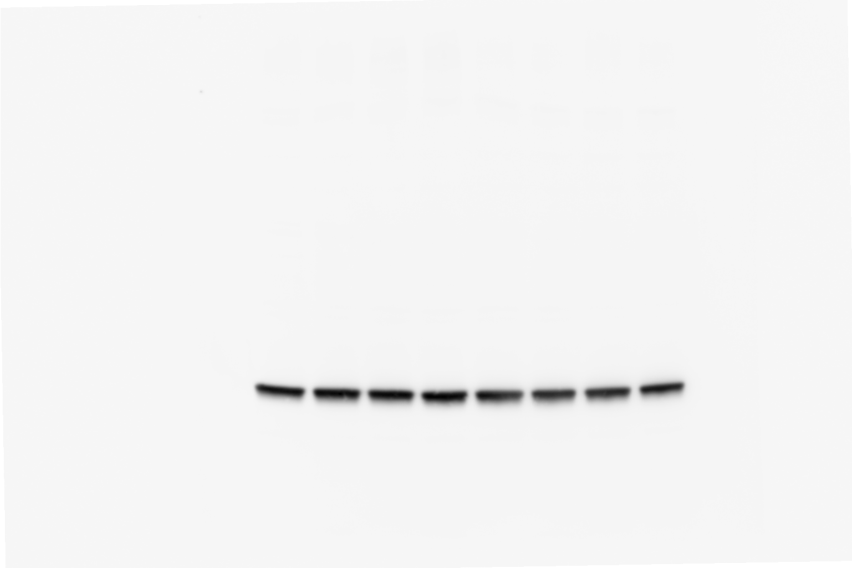

Supplement: Supplementary file 1 — Supplementary information [file 41598_2019_56329_MOESM1_ESM.pdf]
